# Risk Assessment and Cholangiocarcinoma: Diagnostic Management and Artificial Intelligence

**DOI:** 10.3390/biology12020213

**Published:** 2023-01-29

**Authors:** Vincenza Granata, Roberta Fusco, Federica De Muzio, Carmen Cutolo, Francesca Grassi, Maria Chiara Brunese, Igino Simonetti, Orlando Catalano, Michela Gabelloni, Silvia Pradella, Ginevra Danti, Federica Flammia, Alessandra Borgheresi, Andrea Agostini, Federico Bruno, Pierpaolo Palumbo, Alessandro Ottaiano, Francesco Izzo, Andrea Giovagnoni, Antonio Barile, Nicoletta Gandolfo, Vittorio Miele

**Affiliations:** 1 Division of Radiology, Istituto Nazionale Tumori IRCCS Fondazione Pascale—IRCCS di Napoli, 80131 Naples, Italy; 2 Medical Oncology Division, Igea SpA, 80013 Naples, Italy; 3 Diagnostic Imaging Section, Department of Medical and Surgical Sciences & Neurosciences, University of Molise, 86100 Campobasso, Italy; 4 Department of Medicine, Surgery and Dentistry, University of Salerno, 84081 Salerno, Italy; 5 Division of Radiology, Università degli Studi della Campania Luigi Vanvitelli, 80138 Naples, Italy; 6 Radiology Unit, Istituto Diagnostico Varelli, Via Cornelia dei Gracchi 65, 80126 Naples, Italy; 7 Nuclear Medicine Unit, Department of Translational Research, University of Pisa, 56216 Pisa, Italy; 8 Department of Radiology, Careggi University Hospital, Largo Brambilla 3, 50134 Florence, Italy; 9 Department of Clinical, Special and Dental Sciences, University Politecnica delle Marche, Via Conca 71, 60126 Ancona, Italy; 10 Department of Radiology, University Hospital “Azienda Ospedaliera Universitaria delle Marche”, Via Conca 71, 60126 Ancona, Italy; 11 Department of Applied Clinical Sciences and Biotechnology, University of L’Aquila, Via Vetoio 1, 67100 L’Aquila, Italy; 12 SSD Innovative Therapies for Abdominal Metastases, Istituto Nazionale Tumori IRCCS-Fondazione G. Pascale, 80130 Naples, Italy; 13 Division of Epatobiliary Surgical Oncology, Istituto Nazionale Tumori IRCCS Fondazione Pascale—IRCCS di Napoli, 80131 Naples, Italy; 14 Diagnostic Imaging Department, Villa Scassi Hospital-ASL 3, Corso Scassi 1, 16149 Genoa, Italy; 15 Italian Society of Medical and Interventional Radiology (SIRM), SIRM Foundation, Via della Signora 2, 20122 Milan, Italy

**Keywords:** cholangiocarcinoma, surveillance, risk assessment, artificial intelligence, radiomics

## Abstract

**Simple Summary:**

The only curative treatment for intrahepatic cholangiocarcinoma (iCCA) is surgical resection, and an early diagnosis is the most effective way to improve survival. In this context, Artificial Intelligence models may be able to evaluate higher-risk patients and thus improve diagnosis.

**Abstract:**

Intrahepatic cholangiocarcinoma (iCCA) is the second most common primary liver tumor, with a median survival of only 13 months. Surgical resection remains the only curative therapy; however, at first detection, only one-third of patients are at an early enough stage for this approach to be effective, thus rendering early diagnosis as an efficient approach to improving survival. Therefore, the identification of higher-risk patients, whose risk is correlated with genetic and pre-cancerous conditions, and the employment of non-invasive-screening modalities would be appropriate. For several at-risk patients, such as those suffering from primary sclerosing cholangitis or fibropolycystic liver disease, the use of periodic (6–12 months) imaging of the liver by ultrasound (US), magnetic Resonance Imaging (MRI)/cholangiopancreatography (MRCP), or computed tomography (CT) in association with serum CA19-9 measurement has been proposed. For liver cirrhosis patients, it has been proposed that at-risk iCCA patients are monitored in a similar fashion to at-risk HCC patients. The possibility of using Artificial Intelligence models to evaluate higher-risk patients could favor the diagnosis of these entities, although more data are needed to support the practical utility of these applications in the field of screening. For these reasons, it would be appropriate to develop screening programs in the research protocols setting. In fact, the success of these programs reauires patient compliance and multidisciplinary cooperation.

## 1. Background

Cancer is a critical obstacle to the improvement of quality of life, representing a leading cause of death worldwide [1]. The World Health Organization (WHO) report in 2021 [2] has shown that tumors are the first or second leading cause of death before the age of 70 years in 112 of 183 states and ranks third or fourth in a further 23 states.

Intrahepatic cholangiocarcinoma (iCCA) is the second most common primary liver tumor, with a median survival of only 13 months. Worldwide, many countirse have suffered considerable escalation in mortality [3,4,5]. Surgical resection is the only curative treatment; however, at first diagnosis, only one-third of patients are at a sufficiently early stage such that this approach can be taken [6,7,8,9,10]. In addition, surgically treated iCCAs show a critical survival rate that ranges from 14% to 33% [11]. Locally advanced or metastatic tumors can be treated with several chemotherapeutic drugs. However, this approach offers few survival benefits [12]. Presently, a number of personalized treatments have been proposed as second-line therapies, although the choice should be correlated with the identification of a molecular profile [13]. Therefore, several ablative treatments have been assessed, although the real effects on survival should been proven [14,15,16,17,18,19,20,21,22,23,24,25,26].

According to their pathological features, iCCAs are classified as small- or large-duct types [27,28,29,30,31,32,33]. Regarding the small-duct subtype, affected patients present a mass-forming growth and chronic liver diseases are often comorbidities. Large-duct iCCA occurs as an infiltrative adenocarcinoma with a fibrotic stroma; this subtype is correlated with chronic cholangiopathies including primary sclerosing cholangitis and liver flukes. Mutations in KRAS and SMAD4 and the amplification of MDM2 are generally identified in large-duct iCCA; thus, S100P is a good marker for large-duct iCCA [27,28,29,30,31,32,33]. IDH1/2, BAP1, or FGFR2 are typical molecular features of small-duct iCCA, which are measured via C-reactive protein and N-cadherin in affected patients [27,28,29,30,31,32,33]. In addition to the well-known subtypes, unusual subtypes have also been identified, including a tubulocystic variant, a mucoepidermoid variant, an enteroblastic variant, and a cholangioblastic cholangiocarcinoma [27,28,29,30,31,32,33].

Small-duct iCCAs are associated with a better prognosis [27], which may be correlated with therapeutically significant IDH1-/2-mutations and FGFR2 gene fusions [28,29]. Correct patient stratification, according to molecular subgroups, could favor the development of personalized therapeutic strategies. In addition, an early diagnosis remains an effective way to improve overall survival, since a surgical approach is the only curative treatment. Therefore, the identification of higher-risk patients, whose risk is correlated with genetic and pre-cancerous conditions, and the employment of non-invasive-screening modalities would be appropriate. In this narrative review, we report an update on the risk features, screening guidelines, screening modalities, and the role of radiomics concerning patients at risk for iCCA.

## 2. Risk Factors

The precise features leading to the onset of iCCA are unknown, although several modifiable and non-modifiable risk factors are known [9,34,35,36,37,38,39,40,41,42,43,44,45,46,47,48,49,50,51]. With regard to the modifiable risk factors, these include alcohol consumption, dietary factors, smoking, hypertension, and obesity [9]. With regard to the non-modifiable risk factors, the following correlates are known: age, gender, type 2 diabetes mellitus (T2DM), genetic mutations (TERT, TP53, WNT or CTNNB1, KRAS, BRAF, SMAD4, FGFR2, IDH1 and IDH2, ARID1A, ARID2, PBRM1, BAP1, MLL3 or KMT2C, HDAC6, BRCA2, EGFR, and NTRKs), and clinical conditions such as Primary Sclerosing Cholangitis (PSC), biliary tree lithiasis, Cirrhosis, HBV and HCV, infectious Non Alcolic Fatty Liver Disease (NAFLD), and Inflammatory Bowel Disease (IBD) [9]. In this scenario, it is clear that surveillance or preventive procedures would only be useful for a limited portion of patients. Nevertheless, assessing the iCCA rate of at-risk patients is critical since an early diagnosis may imply a better prognosis.

## 3. Primary Sclerosing Cholangitis

**Figure 1:**
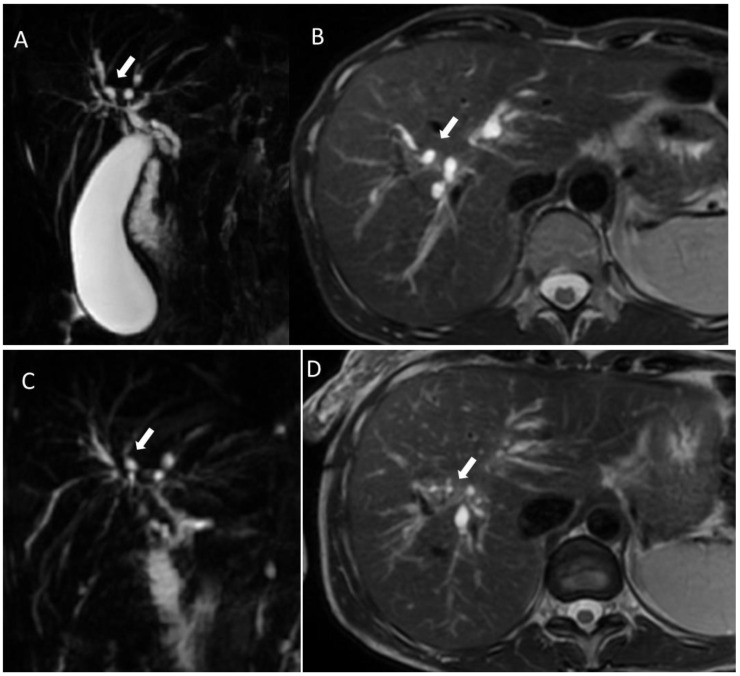
MRI of a 45-year-old patient affected by sclerosing cholangitis at baseline ((**A**): cholangiography sequence; (**B**): T2-weigthed sequence in axial plane) and 6 years later after cholecystectomy ((**C**): cholangiography sequence; (**D**): T2-weigthed sequence in axial plane). (**A**,**C**) 3D Magnetic resonance cholangiopancreatography and (**B**,**D**) T2 Axial images. The figures show progressive strictures and focal dilatations (white arrows) of intrahepatic biliary ducts over a period of 6 years.

Primary sclerosing cholangitis, a chronic cholestatic disease, is characterized by inflammation and fibrosis of the intrahepatic and/or extrahepatic bile ducts. This process, of unknown etiology, is responsible for the development of multifocal strictures and bile duct dilatations (Figure 1). 

PSC is strongly correlated with IBD (about 70–75% of patients), and the presence of both conditions in patients causes an increased risk of hepatobiliary and colorectal cancers [39,40,41,42,43,44,45,46,47,48,49,50,51]. In addition, PSC is the principal risk condition for CCA; thus, CCA remains a significant cause of mortality in patients with PSC [39,40,41,42,43,44,45,46,47,48,49,50,51]. The incidence of CCA in PSC varies between 0.6–1.5%, with a life-time risk of up to 20% [52]. Although CCA can be the first sign of previously undiagnosed PSC, about half of CCA-related PSC are diagnosed within the first year of PSC diagnosis [53,54]. The incidence of tumor development is the highest in patients with dominant strictures compared to small-duct PSC; in 76%, of cases the lesion is located in the peri-hilar region [39,40,41,42,43,44,45,46,47,48,49,50,51].

Today, since no surveillance strategy has been efficient with respect to the early detection of CCA, the clinical utility of surveillance is doubtful. The British Society of Gastroenterology’s UK-PSC guidelines [43], compiled as a result of several study groups [53,54,55,56,57,58], do not suggest routine screening for CCA. However, the Mayo Clinic study group showed that annual surveillance, based on abdominal ultrasound (US), computed tomography (CT), or magnetic resonance imaging (MRI)/cholangiopancreatography (MRCP) plus CA19-9, is correlated with a higher 5-year iCCA-related survival compared to a non-surveillance group (21% vs. 8%) [59].

In addition, the Italian Clinical Practice Guidelines on Cholangiocarcinoma study group [9,10] proposed the use of regular (6–12 months) liver evaluation via US, MRI/MRCP, or CT combined with serum CA19-9 assessment for PSC patients with clinically stable disease.

A position statement from the International PSC Study Group (IPSCSG) [60] proposed MRI/MRCP as the first imaging tool applicable to PSC patients that assesses several prognostic features, such as parenchymal abnormalities, variable enhancement, liver stiffness, and functionality, by utilizing hepatobiliary specific contrast agents to evaluate the sverity of liver fibrosis and the dilatation of the bile duct [61,62,63,64,65,66,67,68,69,70,71,72,73,74,75,76,77,78,79,80,81,82,83].

Regarding the reporting of radiological findings, considering the necessity of using a standardized lexicon [84,85,86,87,88,89,90,91,92,93,94], especially for the early detection of CCA, several studies have proposed the definition of terminologies and reporting standards to describe MRI/MRCP features during PSC patients’ surveillance [39]. According to these studies [39], signs of CCA can be progression in the severity of previously detected strictures and/ or an increasing upstream dilatation. In addition, contrast studies should be considered since any focal nodular thickening enhancement or focal thickening with associated portal vein narrowing should raise suspicion of CCA [39]. Functional studies based on diffusion-weighted imaging (DWI) could offer additional data; bile ducts can demonstrate diffusion restriction in the setting of inflammation and tumors [39,95,96,97,98,99,100,101,102,103,104]. For suspicious structures, an endoscopic retrograde cholangiopancreatography (ERCP) for biliary brushing should be considered [39].

Parenchymal atrophy and/or hypertrophy are non-specific features and can be seen in the setting of PSC or CCA [105,106,107,108,109,110,111,112,113,114,115,116,117,118,119,120,121,122,123,124,125]. The presence of liver metastases, lymphadenopathy, and peritoneal disease should be assessed [105,106,107,108,109,110,111,112,113,114,115,116,117,118,119,120,121,122,123,124,125].

## 4. Fibropolycystic Liver Disease

Fibropolycystic liver disease comprises several conditions, such as congenital hepatic fibrosis, choledocal cysts biliary, Caroli disease, and hamartomas. These afflictions have been proven to lead to a lifetime risk of CCA with an Odds Ratio (OR) similar to PSC [52,126,127].

**Figure 2:**
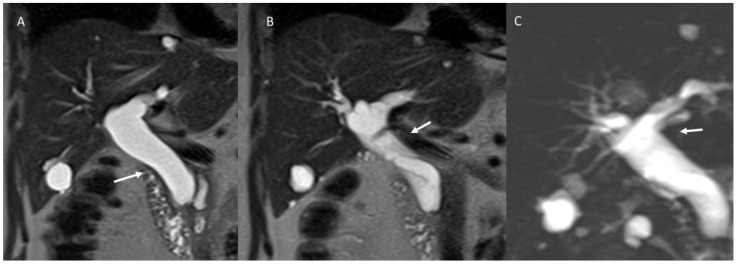
MRI of a 64-year-old male patient with Choledochal cysts. (**A**,**B**): T2-weigthed sequences in coronal plane; (**C**): 3D Magnetic resonance cholangiopancreatography. White arrows show dilatations of the extrahepatic biliary tree.

Choledochal cysts (CCs), which are uncommon in Western populations (incidence of 1 to 100,000–150,000) and more frequent in some Asian populations (incidence of 1 in 1000), are congenital dilatations of the biliary tree, involving either extrahepatic (Figure 2) and/or intrahepatic biliary ducts [128,129,130,131,132,133].

**Figure 3:**
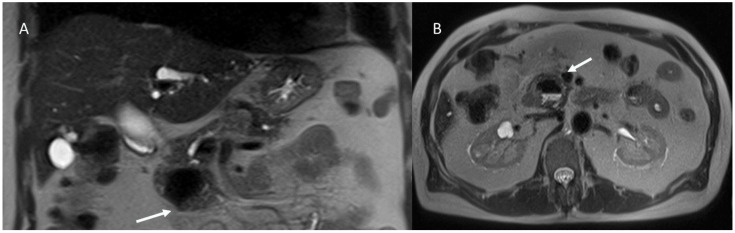
MRI of a 68-year-old male patient with Todani type II lesion. White arrows in coronal (**A**) and axial (**B**) T2-weigthed sequences show an extrahepatic supraduodenal diverticulum.

**Figure 4:**
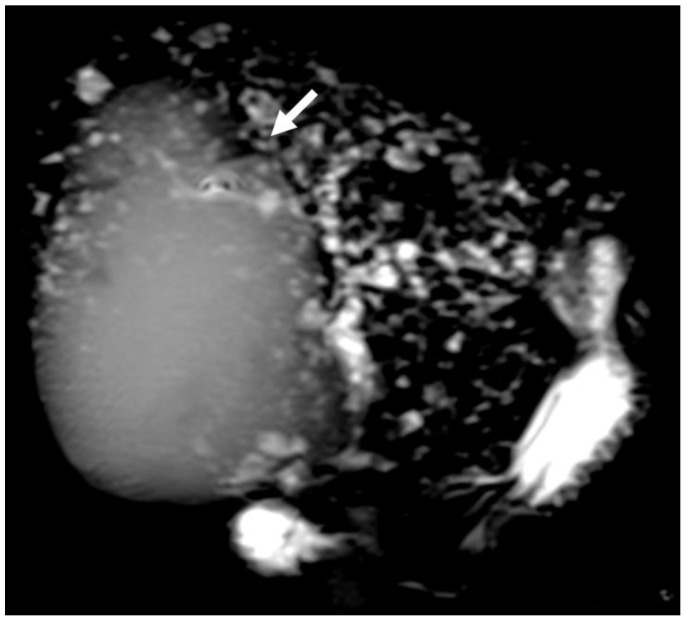
2D Magnetic resonance cholangiopancreatography of 48-year-old female patient with Caroli’s disease (white arrow shows cystic lesion).

CCs are usually categorized according to the Todani classification [134]: type I, the most common, is a solitary extrahepatic cyst; type II is an extrahepatic supraduodenal diverticulum (Figure 3); type III is an intraduodenal cyst (e.g., choledochocele); type IV involves extrahepatic and intrahepatic cysts; and type V, referred to as Caroli’s disease (Figure 4), comprises multiple intrahepatic cysts.

Although CC diagnosis can be accidental, during a liver assessment for other clinical indications, the diagnosis and characterization should be made with MRI/MRCP and ERCP [135].

Since malignancy risk increases with age, an early diagnosis and treatment leads to a more favorable outcome [135]. With regard to the different sub-types and malignancy risk, it has been demonstrated that among patients who develop biliary malignancy, around 68% occurred in type I, around 5% in type II, 1.6% in type III, 21% in type IV, and 6% in type V [134]. In addition, this risk increases if there is an anomalous pancreatic biliary junction (APBJ) [135], which is thought to be correlated with CCs.

Presently, treatment recommendations are based on Asian data, and there is no evidence that suggests an over-treatment of Western patients when Asian guidelines are followed. Therefore, current data suggest comparable efficacy of the treatment options between East and West [135].

With regard to surveillance, according to the Italian Clinical Practice Guidelines on Cholangiocarcinoma study group [9,10], these patients should be subjected to the same PSC patient strategy, comprising periodic (6–12 months) imaging evaluation using US, MRI/MRCP, or Computed tomography (CT) combined with serum CA19-9 measurement.

## 5. Liver Cirrhosis and Hepatitis

Liver cirrhosis is characterized by disseminated fibrosis and regenerative nodules due to chronic liver injury [136]. Cirrhosis can be the result of numerous insults, such as alcohol, NASH, autoimmune or viral hepatitis (B and C virus), and a number of toxic and metabolic reasons [136]. Whatever the cause of cirrhosis, several authors have assessed the iCCA risk in cirrhosis patients [39,40,137,138,139,140,141,142]. A meta-analysis that included 7 case-control studies with 339,608 patients demonstrated that cirrhosis has an OR of 22.9 when coupled with iCCA [143]. According to the European Network for the Study of Cholangiocarcinoma (ENSCCA) [144], among the 2234 analyzed patients, including 1243 (55.6%) with iCCA, 592 (26.5%) with pCCA, and 399 (17.9%) with distal (d)-CCA, 2.8% of them had hepatitis C virus, 4.6% had hepatitis B virus, 0.1% had a concomitant infection (mainly in the iCCA group), and 7.8% had cirrhosis (mainly in iCCA group).

In addition, although HBV and HCV are well known risk factors for cirrhosis, these viral agents have both been associated with approximately two-fold increases in CCA risk, independently of cirrhosis [145].

**Figure 5:**
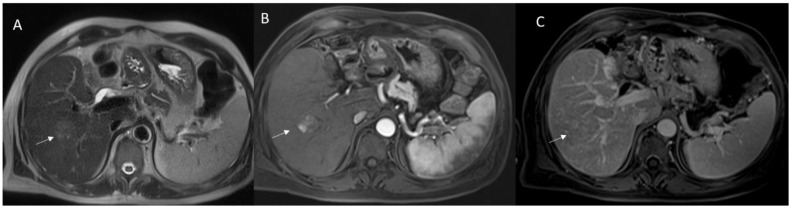
MRI of HCV 47-year-old patient with a case of intrahepatic cholangiocarcinoma. In T2-weigthed sequence (**A**), the lesion (arrow) shows inhomogeneous signal, iso-hyperintense. During vascular assessment ((**B**): arterial phase; (**C**): portal phase), the lesion is characterized as LR-M (rim APHE (in (**B**), white arrow) and without washout (in (**C**), white arrow)). (**B**,**C**): gradient T1-weigthed sequences (Volumetric interpolated breath-holding examination sequence), in axial plane in arterial and portal phases.

Although the relationship between HBV and iCCA is not as strong as for HCC, a meta-analysis and observational studies showed a four-fold to six-fold increase in risk [146,147,148]. Additionally, it is uncertain whether iCCA in HBV-infected patients is correlated with the virus itself or the development of cirrhosis. There are several histo-pathological differences between iCCAs in HBV-infected and uninfected patients that could support the idea of different biological factors and mechanisms that favor the onset of CCA. In fact, iCCA in infected HBV-patient is usually mass forming, with a higher percentage of capsule formation and lower percentage of lymphatic involvement [145]. Compared to HBV, HCV infection is more constantly correlated with a higher risk (Figure 5) [145].

Liver fluke infections are endemic in Korea, Thailand, China, Laos, Vietnam, and Cambodia. CCA is correlated with Opisthorchis viverrini, Opisthorchis felineus, and Clonorchis sinensis infection due to the consumption of raw or undercooked freshwater fish [149]. 

Human Immunodeficiency Virus (HIV) infection may increase the risk of iCCA [145]. HIV is known to be correlated with an increased risk of cholangitis via AIDS cholangiopathy or due to opportunistic infections [145].

With regard to obesity, metabolic syndrome, diabetes, NAFLD, smoking, alcohol, hepatolithiasis, and inflammatory disorders, since all these entities are responsible for chronic inflammation and cirrhosis, it was proposed that these patients should be monitored as is performed for patients with HCC [9,150].

The HCC-screening guidelines developed by the AASLD and the European Association for the Study of the Liver (EASL) and the Asian Pacific Association for the Study of the Liver (APASL) recommend surveillance for HCC in select high-risk populations with US alone (EASL), in concert with AFP (APASL), or with AFP as an option (AASLD), using a surveillance interval of 6 months. Given its remarkable safety, accessibility, and cost-effectiveness, US is the main imaging tool for patients at high risk for HCC [150,151].

The American College of Radiology (ACR) proposed a standardized algorithm for screening and surveillance of high-risk patients using US (US-LI-RADS) [152,153,154,155,156,157,158,159,160,161,162,163,164]. The LI-RADS algorithm provides a standardized lexicon, diagnostic protocol, and a guide to assess several radiological patterns that can be reported to patients at risk for HCC. The system has been updated since the first edition in 2011, with the latest edition released in 2018 [164].

While in CT/ MRI, as in contrast-enhanced (CE) LI-RADS, each single observation is assigned to a category, for US LI-RADS, category assignment is performed in accordance with a full examination by utilizing observations of the main suspected tumors and includes three categories: US-1 negative, US-2 subthreshold, and US-3 positive. According to the assigned category, it has proposed several recommendations [164]:

For US-1, the patient should undergo routine surveillance consisting of a US examination every 6 months.

For the US-2 category, in which a tumor < 10 mm (or more) is measured, it is requested that the nodules are analyzed via US surveillance for up to 2 years with short-intervals (every 3–6-months). 

For US-3 positive assignment, further characterization (HCC vs. iCCA) with multiphase contrast studies is suggested [164].

## 6. Precursors Lesions of Cholangiocarcinoma

CCA Carcinogenesis is a multifarious process starting with transformed biliary epithelial cells or originating from stem/progenitor cells.

### 6.1. Biliary Intraepithelial Neoplasia

Biliary intraepithelial neoplasia (BilIN) is the main common precursor of CCA [165]. BilIN can appear as a non-invasive, microscopic, flat, micropapillary (a papillary projection with a fibrovascular stalk) or pseudopapillary (a papillary projection without a fibrovascular stalk) lesion with dysplasia. Macroscopically, although these entities may present with fine granularity, thickened velvety mucosa, or effacement of underlying tissue layers, in several cases, they can appear as grossly normal [166,167,168]. In addition, multicentricity is common. In 2019, the WHO classified BilIN into two histological entities, namely, those of high and low grade, with high grade categorized as a carcinoma in situ [167]. BilIN is found in hepatolithiasis, PSC and choledochal cysts, and in cirrhotic livers from non-biliary diseases [168]. Usually, BilIN is asymptomatic and not detectable by diagnostic studies [166].

### 6.2. Intraductal Papillary Neoplasia of the Bile Duct

**Figure 6:**
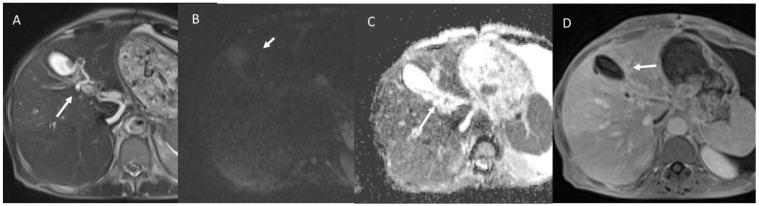
MRI of a 48-year-old female patient with intraductal papillary neoplasms of the bile duct, which were shown to be enlarged in the bile duct (in (**A**): T2-W sequences, white arrow), in which is possible to find mucus. In Diffusion-weighted-imaging assessment ((**B**): b800 s/mm2; (**C**): Apparent Diffusion Map), the lesion (white arrows) shows no restricted diffusion due to mucus. In post-contrast evaluation (in (**D**): Volumetric interpolated breath-holding examination T1-weigthed sequence in portal phase), there is no contrast enhancement (white arrow).

**Figure 7:**
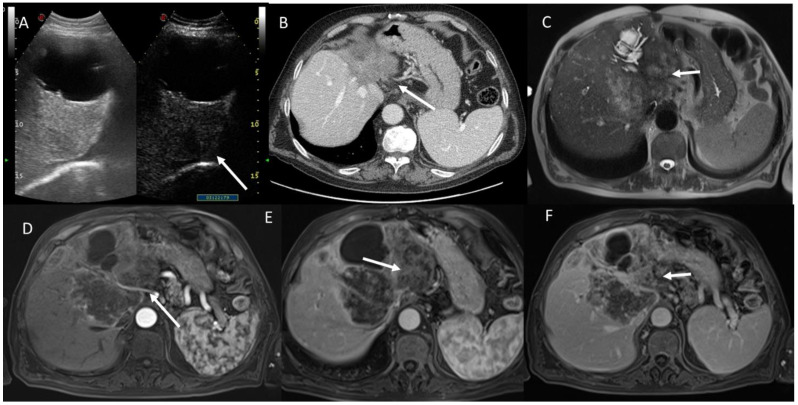
Imaging assessment of degenerated intraductal papillary neoplasms of the bile duct in a 78-year-old patient. (**A**) Contrast-enhanced ultrasound evaluation, with progressive contrast enhancement of invasive lesion component (white arrow). This pattern (white arrow) is also present in CT assessment ((**B**): portal phase) and in MRI contrast study (Volumetric interpolated breath-holding examination sequence in (**D**): arterial phase, (**E**): late arterial phase, and (**F**): portal phase). In T2-weigthed sequences (**C**), the invasive component shows inhomogeneous iso-hyperintense signal (white arrow).

**Figure 8:**
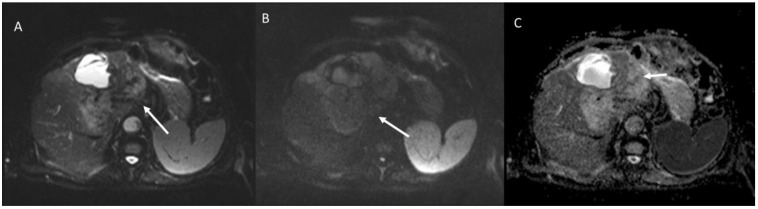
The same patient of Figure 7. Diffusion weighted imaging assessment ((**A**): b50 s/mm^2^; (**B**): b800 s/mm^2^; (**C**): Apparent Diffusion Coefficient map) the lesion shows restricted diffusion (white arrows in (**A**,**B**)) and targetoid appearance in apparent diffusion coefficient map (white arrow in (**C**)).

Intraductal papillary neoplasia of the bile duct (IPNB) is a macroscopic premalignant lesion (Figure 6) that may arise within intra- or extrahepatic bile ducts [169,170,171,172,173,174]. Macroscopically, IPNBs present as detectable papillary, polypoid, greyish brown or white, soft tissue growths inside an enlarged bile duct, in which it is possible to find mucus. Like pancreatic intraductal papillary mucinous neoplasm (IPMN), IPNB is histologically classified into four types: intestinal, pancreatic biliary, gastric, and oncocytic subtypes [170,171,172,173,174]. Intestinal and pancreatic biliary are the most common subtypes, and high-grade dysplasia is usually massive, thus signalling the presence of invasive cancer (Figure 7 and Figure 8) in approximately half of cases. Therefore, surgical resection is suggested for this entity [170].

IPNB is correlated with PSC, congenital biliary tract disease, hepatolithiasis, and liver fluke infections. These tumors, single or multiple, can cause large duct obstruction with abdominal pain, cholangitis, and cholestatic hepatic dysfunction [171].

### 6.3. Intraductal Tubulopapillary Neoplasms of the Bile Duct

Intraductal tubule-papillary neoplasms of the bile duct (ITPNs) constitute a new entity characterized by a tubular growth pattern in the large intrahepatic and extrahepatic bile ducts that is associated with invasive cancer at the diagnosis [171]. ITPNs appear as a polypoid or solid tumor inside a dilated bile duct [172]. Compared to IPNB, ITPNs have a better prognosis [173,174].

### 6.4. Hepatobiliary Mucinous Cystic Neoplasm

**Figure 9:**
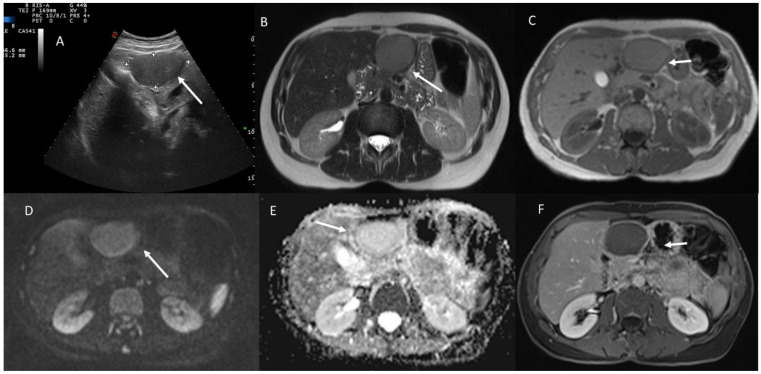
US (**A**) and MRI (**B**–**F**) assessment of cystadenoma patient. Upon US (**A**), the lesion shows solid appearance (white arrow), while in T2-weigthed sequences (**B**) and T1-weighted imaging (**C**): in phase) it shows non-solid pattern (white arrows) with restricted diffusion (**D**): b800 s/mm^2^ and (**E**): Apparent Diffusion Coefficient map) and peripheral contrast enhancement (white arrow) in T1-weigthed sequence in portal phase of contrast study.

**Figure 10:**
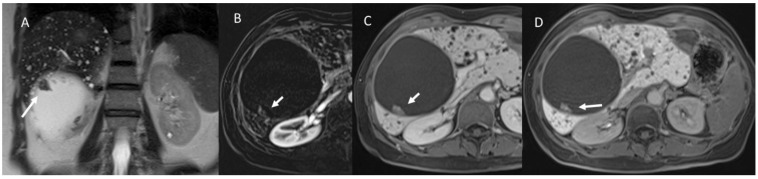
MRI assessment of female cystadenocarcinoma patient. The white arrows show papillary soft tissue growths inside cystic lesion with solid appearance in T2-weigthed (**A**) and progressive contrast enhancement during arterial (**B**) (subtract phase), portal (**C**), and liver-specific phase (**D**) using T1-weigthed post contrast sequences (volumetric interpolated breath-holding examination sequence).

Hepatobiliary mucinous cystic neoplasms (HMCNs), previously defined as cystadenoma (Figure 9) or cystadenocarcinoma (Figure 10), include neoplastic mucinous and/or non-mucinous biliary epithelia surrounded by ovarian-type mesenchymal stroma [175]. This entity is rare, representing less than 5% of all hepatic cystic neoplasms that are detected in women during the fourth or fifth decade of life [176,177,178]. 

HMCNs have either low-grade dysplasia or malignant features with high-grade dysplasia [179].

### 6.5. Diagnostic Management

For the detection of lesions with MRI combined with a functional assessment, including a DWI, the combined use of a hepatospecific contrast agent and cholangiography sequences constitute an optimal modality since it allows for the assessment of the lesion and its relationship with the biliary branches and the contiguous hepatic parenchyma [180,181], thereby permitting proper treatment planning.

Since almost all of these precursor lesions are found in patients at risk for iCCA, such as those with PSC, congenital biliary tract disease, hepatolithiasis, and liver fluke infections, and as surveillance protocols with periodic (6–12 months) imaging of the liver by US, MRI/MRCP, or CT combined with serum CA19-9 measurement have been proposed for several groups [9,10], it is clear that for all newly detected lesions inside a biliary duct, a proper multidisciplinary evaluation should be proposed. Although these precursor lesions are rare entities, the possibility of characterizing them in the early stage facilitates the improvement of patient survival and reduces costs due to invasive procedures that could affect patients’ quality of life. In addition, it is evident that these conditions should be managed in dedicated and expert groups including surgeons, radiologists, pathologists, and molecular biologists to avoid unnecessary treatment and to achieve improved patient management [180]. With regard to imaging tools, although CT and US are the main modalities and are often the first tools employed, for a correct characterization and evaluation of bile ducts, MRI should be performed for all doubtful cases. To the best of our knowledge, no standardized protocols have been suggested; however, it is critical that these patients are assessed in cancer centers [180].

## 7. Artificial Intelligence, Radiomics, and Cholangiocarcinoma

The latest scientific developments have improved the use of artificial intelligence (AI) in the medical setting. Since computers can accumulate and assess higher volumes of data compared to the human brain, AI can resolve difficulties in the oncological setting [182,183,184,185,186,187,188,189,190,191,192,193,194,195,196,197,198]. AI is a branch of computer science that develops algorithms trained to execute functions that are normally performed by the human brain [198,199,200,201,202,203,204,205,206,207,208,209,210]. Machine learning (ML), a sub-area of AI, is based on computer models that can learn specific tasks through the replication of computations resulting from considerable volumes of data [211,212,213,214,215,216,217,218,219,220,221,222,223,224,225,226,227,228]. These models evaluate data using mathematical algorithms that are frequently corrected until the analysis yields the required outcome. These models can be supervised or unsupervised, and their applicational diversity correspoinds to the computerized knowledge of the desired outcome of interest [229,230,231,232,233,234,235,236,237,238]. In the supervised form, a training dataset (the “input”) is introduced to obtain the desired outcome (the “output”). Therefore, the ML model analyzes the input, producing the necessary adjustments to obtain the desired output [239,240,241,242,243,244,245,246,247,248,249,250,251]. This type of learning requires great great volumes of training data that have been “curated”, that is, pre-labeled by a human operator. Once the training is completed, a different dataset (testing data) is used to test the model’s performance [252,253,254,255,256,257,258,259,260]. In unsupervised learning, the model assesses un-curated data, classifying the data due to defined features within the dataset that can be grouped and analyzed further to reach a specific outcome [261,262,263,264,265,266,267,268,269,270,271,272,273,274,275,276,277,278,279,280].

**Figure 11:**
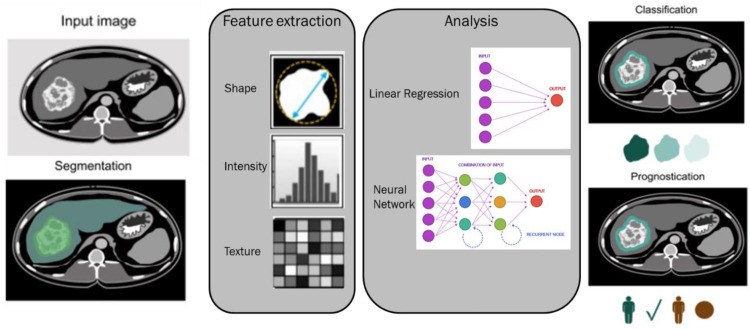
Flowchart of artificial intelligence model.

To date, many of the ML models utilized in the clinical setting have been supervised, and these models have enabled the development of a new approach named Radiomics [281,282,283,284,285,286,287,288,289,290,291]. Radiomics is based on the extraction by digital images of data that can be analyzed in a mathematical way [281,282,283,284,285,286,287,288,289,290,291]. The idea that imaging studies contain a great quantity of data, in the form of grey-level patterns, which are imperceptible to the human eye, has become increasingly interesting [292,293,294]. These texture features, when correlated with clinical-pathological data and outcomes, theoretically allow for diagnostic and prognostic assessment [295,296,297,298] (Figure 11 reports a typical flowchart of an artificial intelligence model).

The assessment of textural characteristics, obtained by conventional radiological images, such as CT or MRI, allow for the extraction of biological data using a non-invasive approach, thereby reducing costs and time and avoiding any risk for the patients. For several tumors, radiomics analyses have already provided accurate evaluations of their biology, thus allowing for the identification of features correlated with clinical outcomes [299,300,301,302,303,304,305,306].

The merit of this new tool is that it is able to obtain digital data from medical imaging and, when performed under appropriate protocols, is more robust and reproducible. Nevertheless, there are remaining issues in the clinical setting. First, reproducibility is a very important issue. This is correlated with several features, such as the acquisition protocol, the method of segmentation, the method for extracting the imaging features, and the acquisition of clinical and genomic data.

In the context of iCCA, the possibility to identify a lesion at an early stage or in a pre-malignant setting may allow for its proper management [307]. Therefore, several researchers have evaluated AI in the CCA setting.

Only few authors have evaluated the possibility of assessing iCCA at an early phase. Xu et al. [308] established a support vector machine (SVM) based on radiomics features obtained by non-enhanced CT to train a discriminative model to recognize HCC and ICCA at an early stage. They showed that compared to radiologists, their model offered significantly better performance with respect to distinguishing HCC from ICCA. Ichikawa et al. [309] obtained similar results.

Logeswaran et al. [310] proposed a CAD system, the multi-layer perceptron (MLP), to automatically detect CCA using a single MRCP image. MLP, a form of ANN, was employed to distinguish patients with and without CCA. Comparing this AI system to previously available systems, MLP was found to be better in terms of the detection of CCA (88.03% vs. 86.17%), healthy tissue (83.64% vs. 76.90%), and non-cholangiocarcinoma lesions (90.14% vs. 80.99%) [310].

Future studies on the possibility of identifying the precursors of CCA, incorporating the ability to evaluate liver parenchyma in which a lesion is not yet visible to the human eye, are desirable. This would allow for the definition of diagnostic-therapeutic programs centered on a single patient.

## 8. Discussion

The primary end point of cancer screening is to reduce tumor-related mortality by detecting cancer at an early stage and preventing its occurrence by identifying and treating pre-malignant lesions in asymptomatic patients [200]. However, at the time of diagnosis, iCCA is often metastatic or in a locally advanced stage. So, the identification of higher-risk patients, defined by genetic and predisposing diseases, using appropriate diagnostic tools is desirable [200]. 

However, the potential hazards of screening programs comprise adverse events associated with diagnostic procedures and patient anxiety [132]. In addition, potential over-diagnosis or misdiagnosis may occur, causing an over-treatment of completely benign or low-risk neoplastic lesions [132].

Today, in the iCCA context, proper surveillance protocols are only proposed for a few categories, such as PSC or cirrhosis patients, although, in this case the pre-existing programs are designed for HCC risk. So, due to the incidence and high mortality rate of iCCA, which are also correlated with a late diagnosis, dedicated programs of screening should be considered. In addition, knowledge of the precursor lesions is critical to avoid a misdiagnosis. In fact, the major limitation is the incapacity to detect and characterize premalignant lesions. The possibility to utilize AI models to evaluate higher-risk patients could benefit the diagnosis of these entities, although more data are needed to support the practical utility of these applications in the field of screening. 

At present, with regard to iCCA, the main clinical setting in which radiomics is employed is treatment, either proper treatment selection such as ablation therapy [311] or for early recurrence, e.g., after surgical resection [312,313,314,315,316]. However, these applications correspond to a late disease status, whereas the ability to identify precursor lesions as soon as possible should improve overall survival. AI allows for the performance of the image-based, extensive analysis of such minute alterations and the identification of potential risk predictors for disease. AI systems, as opposed to manual approaches, execute complex tasks without interruption and ensure highly accurate and precise outcomes. In the domain of the automated processing and analysis of medical images, AI offers numerous techniques and tools with which to extract accurate measurements from different structures, and can identify nonlinear features and evaluate tissue properties. For prediction modelling, radiomics analysis as well as machine and deep learning are regarded as the most reliable and common AI approaches. The main issues remain those related to the dataset employed, which should include a large number of data, so that, in rare conditions, even few results can be robust and reproducible.

Accordingly, it would be appropriate to realize screening programs in the research protocols setting. In fact, the success of these programs requires patient compliance and multidisciplinary cooperation [132].

## 9. Conclusions

Although the knowledge on iCCA is increasing, currently, its diagnosis in a late stage is correlated with a high rate of patients that are unfit for surgical treatment. Dedicated surveillance programs have been proposed for patients in a few at-risk categories. Improved stratification of patients accodgin to the underlying liver disease and the introduction of AI systems and Radiomics models may lead to better patient management, which should include a multidisciplinary team of experts and a dedicated study protocol to optimize time, personnel, and economic resources.

## Data Availability

All data are available in the manuscript and at link https://zenodo.org/record/7576415#.Y9PrT3bMK3A (accessed on 1 November 2022).

## References

[1] Sung H., Ferlay J., Siegel R.L., Laversanne M., Soerjomataram I., Jemal A., Bray F. (2021). Global Cancer Statistics 2020: GLOBOCAN Estimates of Incidence and Mortality Worldwide for 36 Cancers in 185 Countries. CA Cancer J. Clin..

[2] https://www.who.int.

[3] Rumgay H., Arnold M., Ferlay J., Lesi O., Cabasag C.J., Vignat J., Laversanne M., McGlynn K.A., Soerjomataram I. (2022). Global burden of primary liver cancer in 2020 and predictions to 2040. J. Hepatol..

[4] Bertuccio P., Malvezzi M., Carioli G., Hashim D., Boffetta P., El-Serag H.B., La Vecchia C., Negri E. (2019). Global trends in mortality from intrahepatic and extrahepatic cholangiocarcinoma. J. Hepatol..

[5] Granata V., Fusco R., Catalano O., Avallone A., Leongito M., Izzo F., Petrillo A. (2017). Peribiliary liver metastases MR findings. Med. Oncol..

[6] Rizvi S., Khan S.A., Hallemeier C.L., Kelley R.K., Gores G.J. (2018). Cholangiocarcinoma—Evolving concepts and therapeutic strategies. Nat. Rev. Clin. Oncol..

[7] Granata V., Grassi R., Fusco R., Setola S.V., Belli A., Ottaiano A., Nasti G., La Porta M., Danti G., Cappabianca S. (2021). Intrahepatic cholangiocarcinoma and its differential diagnosis at MRI: How radiologist should assess MR features. Radiol. Med..

[8] Sardanelli F., Colarieti A. (2022). Open issues for education in radiological research: Data integrity, study reproducibility, peer-review, levels of evidence, and cross-fertilization with data scientists. Radiol. Med..

[9] Cholangiocarcinoma Working Group (2020). Italian Clinical Practice Guidelines on Cholangiocarcinoma-Part I: Classification, diagnosis and staging. Dig. Liver Dis..

[10] Cholangiocarcinoma Working Group (2020). Italian Clinical Practice Guidelines on Cholangiocarcinoma-Part II: Treatment. Dig. Liver Dis..

[11] Xing H., Tan B., Yang C., Zhang M. (2022). Incidence Trend and Competing Risk Analysis of Patients with Intrahepatic Cholangiocarcinoma: A Population-Based Study. Front. Med..

[12] Lamarca A., Palmer D.H., Wasan H.S., Ross P.J., Ma Y.T., Arora A., Falk S., Gillmore R., Wadsley J., Patel K. (2019). ABC-06|A randomised phase III, multi-centre, open-label study of active symptom control (ASC) alone or ASC with oxaliplatin/5-FU chemotherapy (ASC + mFOLFOX) for patients (pts) with locally advanced/metastatic biliary tract cancers (ABC) previously- treated with cisplatin/gemcitabine (CisGem) chemotherapy. J. Clin. Oncol..

[13] Massironi S., Pilla L., Elvevi A., Longarini R., Rossi R.E., Bidoli P., Invernizzi P. (2020). New and Emerging Systemic Therapeutic Options for Advanced Cholangiocarcinoma. Cells.

[14] Patrone R., Izzo F., Palaia R., Granata V., Nasti G., Ottaiano A., Pasta G., Belli A. (2021). Minimally invasive surgical treatment of intrahepatic cholangiocarcinoma: A systematic review. World J. Gastrointest. Oncol..

[15] Granata V., Fusco R., D’Alessio V., Simonetti I., Grassi F., Silvestro L., Palaia R., Belli A., Patrone R., Piccirillo M. (2023). Percutanous Electrochemotherapy (ECT) in Primary and Secondary Liver Malignancies: A Systematic Review. Diagnostics.

[16] Izzo F., Granata V., Fusco R., D’Alessio V., Petrillo A., Lastoria S., Piccirillo M., Albino V., Belli A., Tafuto S. (2021). Clinical Phase I/II Study: Local Disease Control and Survival in Locally Advanced Pancreatic Cancer Treated with Electrochemotherapy. J. Clin. Med..

[17] Izzo F., Granata V., Fusco R., D’Alessio V., Petrillo A., Lastoria S., Piccirillo M., Albino V., Belli A., Nasti G. (2021). A Multicenter Randomized Controlled Prospective Study to Assess Efficacy of Laparoscopic Electrochemotherapy in the Treatment of Locally Advanced Pancreatic Cancer. J. Clin. Med..

[18] Bimonte S., Leongito M., Barbieri A., Del Vecchio V., Barbieri M., Albino V., Piccirillo M., Amore A., Di Giacomo R., Nasto A. (2015). Inhibitory effect of (-)-epigallocatechin-3-gallate and bleomycin on human pancreatic cancer MiaPaca-2 cell growth. Infect. Agent Cancer..

[19] Granata V., Grassi R., Fusco R., Belli A., Palaia R., Carrafiello G., Miele V., Grassi R., Petrillo A., Izzo F. (2021). Local ablation of pancreatic tumors: State of the art and future perspectives. World J. Gastroenterol..

[20] Izzo F., Piccirillo M., Albino V., Palaia R., Belli A., Granata V., Setola S., Fusco R., Petrillo A., Orlando R. (2013). Prospective screening increases the detection of potentially curable hepatocellular carcinoma: Results in 8,900 high-risk patients. HPB.

[21] Argalia G., Tarantino G., Ventura C., Campioni D., Tagliati C., Guardati P., Kostandini A., Marzioni M., Giuseppetti G.M., Giovagnoni A. (2021). Shear wave elastography and transient elastography in HCV patients after direct-acting antivirals. Radiol. Med..

[22] Giovagnoni A. (2021). A farewell from the “old” Editor-in-Chief. Radiol. Med..

[23] Cicero G., Mazziotti S., Silipigni S., Blandino A., Cantisani V., Pergolizzi S., D’Angelo T., Stagno A., Maimone S., Squadrito G. (2021). Dual-energy CT quantification of fractional extracellular space in cirrhotic patients: Comparison between early and delayed equilibrium phases and correlation with oesophageal varices. Radiol. Med..

[24] Granata V., Fusco R., Salati S., Petrillo A., Di Bernardo E., Grassi R., Palaia R., Danti G., La Porta M., Cadossi M. (2021). A Systematic Review about Imaging and Histopathological Findings for Detecting and Evaluating Electroporation Based Treatments Response. Int. J. Environ. Res. Public Health..

[25] Granata V., Grassi R., Fusco R., Setola S.V., Palaia R., Belli A., Miele V., Brunese L., Grassi R., Petrillo A. (2020). Assessment of Ablation Therapy in Pancreatic Cancer: The Radiologist’s Challenge. Front Oncol..

[26] Granata V., Fusco R., Setola S.V., Avallone A., Palaia R., Grassi R., Izzo F., Petrillo A. (2020). Radiological assessment of secondary biliary tree lesions: An update. J. Int. Med. Res..

[27] Nagtegaal I.D., Odze R.D., Klimstra D., Paradis V., Rugge M., Schirmacher P., Washington K.M., Carneiro F., Cree I.A., WHO Classification of Tumours Editorial Board (2020). The 2019 WHO classification of tumours of the digestive system. Histopathology.

[28] Akita M., Fujikura K., Ajiki T., Fukumoto T., Otani K., Azuma T., Itoh T., Ku Y., Zen Y. (2017). Dichotomy in intrahepatic cholangiocarcinomas based on histologic similarities to hilar cholangiocarcinomas. Mod. Pathol..

[29] Saha S.K., Parachoniak C.A., Ghanta K.S., Fitamant J., Ross K.N., Najem M.S., Gurumurthy S., Akbay E.A., Sia D., Cornella H. (2014). Mutant IDH inhibits HNF-4α to block hepatocyte differentiation and promote biliary cancer. Nature.

[30] Kendall T., Verheij J., Gaudio E., Evert M., Guido M., Goeppert B., Carpino G. (2019). Anatomical, histomorphological and molecular classification of cholangiocarcinoma. Liver Int..

[31] Komuta M. (2021). Histological Heterogeneity of Primary Liver Cancers: Clinical Relevance, Diagnostic Pitfalls and the Pathologist’s Role. Cancers.

[32] ASA Physical Status Classification System. https://www.asahq.org/standards-and-guidelines/asa-physical-status-classification-system.

[33] Liau J.Y., Tsai J.H., Yuan R.H., Chang C.N., Lee H.J., Jeng Y.M. (2014). Morphological subclassification of intrahepatic cholangiocarcinoma: Etiological, clinicopathological, and molecular features. Mod. Pathol..

[34] Hemminki K., Sundquist K., Sundquist J., Försti A., Liska V., Hemminki A., Li X. (2022). Personal comorbidities and their subsequent risks for liver, gallbladder and bile duct cancers. Int. J. Cancer..

[35] Donati M., Šteiner P., Kazakov D.V. (2023). BAP1-Inactivated Melanoma Arising From BAP1-Inactivated Melanocytic Tumor in a Patient With BAP1 Germline Mutation: A Case Report and Review of the Literature. Am. J. Dermatopathol..

[36] Uson Junior P.L., Kunze K.L., Golafshar M.A., Riegert-Johnson D., Boardman L., Borad M.J., Ahn D., Sonbol M.B., Faigel D.O., Fukami N. (2022). Germline Cancer Susceptibility Gene Testing in Unselected Patients with Hepatobiliary Cancers: A Multi-Center Prospective Study. Cancer Prev. Res..

[37] Granata V., Fusco R., Catalano O., Avallone A., Palaia R., Botti G., Tatangelo F., Granata F., Cascella M., Izzo F. (2017). Diagnostic accuracy of magnetic resonance, computed tomography and contrast enhanced ultrasound in radiological multimodality assessment of peribiliary liver metastases. PLoS ONE.

[38] Samadder N.J., Riegert-Johnson D., Boardman L., Rhodes D., Wick M., Okuno S., Kunze K.L., Golafshar M., Uson P.L.S., Mountjoy L. (2021). Comparison of Universal Genetic Testing vs Guideline-Directed Targeted Testing for Patients With Hereditary Cancer Syndrome. JAMA Oncol..

[39] Venkatesh S.K., Welle C.L., Miller F.H., Jhaveri K., Ringe K.I., Eaton J.E., Bungay H., Arrivé L., Ba-Ssalamah A., Grigoriadis A. (2022). Reporting standards for primary sclerosing cholangitis using MRI and MR cholangiopancreatography: Guidelines from MR Working Group of the International Primary Sclerosing Cholangitis Study Group. Eur. Radiol..

[40] Eliasson J., Lo B., Scramm C., Chazouilleres O., Folseraas T., Beuers U., Ytting H. (2022). Survey uncovering variations in the management of primary sclerosing cholangitis across Europe. JHEP Rep..

[41] Villard C., Friis-Liby I., Rorsman F., Said K., Warnqvist A., Cornillet M., Kechagias S., Nyhlin N., Werner M., Janczewska I. (2022). Prospective surveillance for cholangiocarcinoma in unselected individuals with primary sclerosing cholangitis. J. Hepatol..

[42] Hoyos S., Navas M.C., Restrepo J.C., Botero R.C. (2018). Current controversies in cholangiocarcinoma. Biochim. Biophys. Acta Mol. Basis Dis..

[43] Chapman M.H., Thorburn D., Hirschfield G.M., Webster G.G.J., Rushbrook S.M., Alexander G., Collier J., Dyson J.K., Jones D.E., Patanwala I. (2019). British Society of Gastroenterology and UK-PSC guidelines for the diagnosis and management of primary sclerosing cholangitis. Gut.

[44] Lieshout R., Kamp E.J.C.A., Verstegen M.M.A., Doukas M., Dinjens W.N.M., Köten K., IJzermans J.N.M., Bruno M.J., Peppelenbosch M.P., van der Laan L.J.W. (2022). Cholangiocarcinoma cell proliferation is enhanced in primary sclerosing cholangitis: A role for IL-17A. Int. J. Cancer.

[45] Fung B.M., Tabibian J.H. (2020). Cholangiocarcinoma in patients with primary sclerosing cholangitis. Curr. Opin. Gastroenterol..

[46] Fong Z.V., Brownlee S.A., Qadan M., Tanabe K.K. (2021). The Clinical Management of Cholangiocarcinoma in the United States and Europe: A Comprehensive and Evidence-Based Comparison of Guidelines. Ann. Surg. Oncol..

[47] Aabakken L., Karlsen T.H., Albert J., Arvanitakis M., Chazouilleres O., Dumonceau J.M., Färkkilä M., Fickert P., Hirschfield G.M., Laghi A. (2017). Role of endoscopy in primary sclerosing cholangitis: European Society of Gastrointestinal Endoscopy (ESGE) and European Association for the Study of the Liver (EASL) Clinical Guideline. Endoscopy.

[48] Fusco R., Simonetti I., Ianniello S., Villanacci A., Grassi F., Dell’Aversana F., Grassi R., Cozzi D., Bicci E., Palumbo P. (2022). Pulmonary Lymphangitis Poses a Major Challenge for Radiologists in an Oncological Setting during the COVID-19 Pandemic. J. Pers. Med..

[49] Tafuto S., von Arx C., De Divitiis C., Maura C.T., Palaia R., Albino V., Fusco R., Membrini M., Petrillo A., Granata V. (2015). ENETS Center of Excellence Multidisciplinary Group for Neuroendocrine Tumors in Naples (Italy). Electrochemotherapy as a new approach on pancreatic cancer and on liver metastases. Int. J. Surg..

[50] Hirschfield G.M., Chazouillères O., Cortez-Pinto H., Macedo G., de Lédinghen V., Adekunle F., Carbone M. (2021). A consensus integrated care pathway for patients with primary biliary cholangitis: A guideline-based approach to clinical care of patients. Expert Rev. Gastroenterol. Hepatol..

[51] Granata V., Fusco R., Setola S.V., Palaia R., Albino V., Piccirillo M., Grimm R., Petrillo A., Izzo F. (2019). Diffusion kurtosis imaging and conventional diffusion weighted imaging to assess electrochemotherapy response in locally advanced pancreatic cancer. Radiol. Oncol..

[52] Clements O., Eliahoo J., Kim J.U., Taylor-Robinson S.D., Khan S.A. (2020). Risk factors for intrahepatic and extrahepatic cholangiocarcinoma: A systematic review and meta-analysis. J. Hepatol..

[53] Boberg K.M., Bergguist A., Mitchell S., Pares A., Rosina F., Broome U., Chapman R., Fausa O., Egeland T., Rocca G. (2002). Cholangiocarcinoma in primary sclerosing cholangitis: Risk factors and clinical presentation. Scand. J. Gastroenterol..

[54] Lazaridis K.N., LaRusso N.F. (2016). Primary Sclerosing Cholangitis. N. Engl. J. Med..

[55] EASL Clinical Practice Guidelines (2009). Management of cholestatic liver diseases. J. Hepatol..

[56] Arslan A., Aktas E., Sengul B., Tekin B. (2021). Dosimetric evaluation of left ventricle and left anterior descending artery in left breast radiotherapy. Radiol. Med..

[57] Haugk B. (2010). Pancreatic intraepithelial neoplasia-can we detect early pancreatic cancer?. Histopathology.

[58] Giurazza F., Cionfoli N., Paladini A., Vallone M., Corvino F., Teodoli L., Moramarco L., Quaretti P., Catalano C., Niola R. (2022). PHIL^®^ (precipitating hydrophobic injectable liquid): Retrospective multicenter experience on 178 patients in peripheral embolizations. Radiol. Med..

[59] Rizvi S., Eaton J.E., Gores G.J. (2015). Primary Sclerosing Cholangitis as a Premalignant Biliary Tract Disease: Surveillance and Management. Clin. Gastroenterol. Hepatol..

[60] Schramm C., Eaton J., Ringe K.I., Venkatesh S., Yamamura J. (2017). MRI working group of the IPSCSG Recommendations on the use of magnetic resonance imaging in PSC-A position statement from the International PSC Study Group. Hepatology.

[61] Granata V., Fusco R., Catalano O., Setola S.V., de Lutio di Castelguidone E., Piccirillo M., Palaia R., Grassi R., Granata F., Izzo F. (2016). Multidetector computer tomography in the pancreatic adenocarcinoma assessment: An update. Infect. Agent Cancer.

[62] Granata V., Fusco R., Venanzio Setola S., Mattace Raso M., Avallone A., De Stefano A., Nasti G., Palaia R., Delrio P., Petrillo A. (2019). Liver radiologic findings of chemotherapy-induced toxicity in liver colorectal metastases patients. Eur. Rev. Med. Pharmacol. Sci..

[63] Granata V., Fusco R., Avallone A., Cassata A., Palaia R., Delrio P., Grassi R., Tatangelo F., Grazzini G., Izzo F. (2020). Abbreviated MRI protocol for colorectal liver metastases: How the radiologist could work in pre surgical setting. PLoS ONE.

[64] Granata V., Fusco R., Petrillo A. (2021). Additional Considerations on Use of Abbreviated Liver MRI in Patients with Colorectal Liver Metastases. AJR Am. J. Roentgenol..

[65] Granata V., Fusco R., Avallone A., Catalano O., Piccirillo M., Palaia R., Nasti G., Petrillo A., Izzo F. (2018). A radiologist’s point of view in the presurgical and intraoperative setting of colorectal liver metastases. Future Oncol..

[66] Granata V., Fusco R., Maio F., Avallone A., Nasti G., Palaia R., Albino V., Grassi R., Izzo F., Petrillo A. (2019). Qualitative assessment of EOB-GD-DTPA and Gd-BT-DO3A MR contrast studies in HCC patients and colorectal liver metastases. Infect. Agent. Cancer..

[67] Granata V., Fusco R., Setola S.V., Piccirillo M., Leongito M., Palaia R., Granata F., Lastoria S., Izzo F., Petrillo A. (2017). Early radiological assessment of locally advanced pancreatic cancer treated with electrochemotherapy. World J. Gastroenterol..

[68] Bimonte S., Leongito M., Granata V., Barbieri A., Del Vecchio V., Falco M., Nasto A., Albino V., Piccirillo M., Palaia R. (2016). Electrochemotherapy in pancreatic adenocarcinoma treatment: Pre-clinical and clinical studies. Radiol. Oncol..

[69] Stefanini M., Simonetti G. (2022). Interventional Magnetic Resonance Imaging Suite (IMRIS): How to build and how to use. Radiol. Med..

[70] Granata V., de Lutio di Castelguidone E., Fusco R., Catalano O., Piccirillo M., Palaia R., Izzo F., Gallipoli A.D., Petrillo A. (2016). Irreversible electroporation of hepatocellular carcinoma: Preliminary report on the diagnostic accuracy of magnetic resonance, computer tomography, and contrast-enhanced ultrasound in evaluation of the ablated area. Radiol. Med..

[71] Barretta M.L., Catalano O., Setola S.V., Granata V., Marone U., D’Errico Gallipoli A. (2011). Gallbladder metastasis: Spectrum of imaging findings. Abdom. Imaging.

[72] Ierardi A.M., Stellato E., Pellegrino G., Bonelli C., Cellina M., Renzulli M., Biondetti P., Carrafiello G. (2022). Fluid-dynamic control microcatheter used with glue: Preliminary experience on its feasibility and safety. Radiol. Med..

[73] Granata V., Fusco R., Piccirillo M., Palaia R., Petrillo A., Lastoria S., Izzo F. (2015). Electrochemotherapy in locally advanced pancreatic cancer: Preliminary results. Int. J. Surg..

[74] Granata V., Fusco R., Setola S.V., Castelguidone E.L.D., Camera L., Tafuto S., Avallone A., Belli A., Incollingo P., Palaia R. (2019). The multidisciplinary team for gastroenteropancreatic neuroendocrine tumours: The radiologist’s challenge. Radiol. Oncol..

[75] Danti G., Flammia F., Matteuzzi B., Cozzi D., Berti V., Grazzini G., Pradella S., Recchia L., Brunese L., Miele V. (2021). Gastrointestinal neuroendocrine neoplasms (GI-NENs): Hot topics in morphological, functional, and prognostic imaging. Radiol. Med..

[76] Chiti G., Grazzini G., Flammia F., Matteuzzi B., Tortoli P., Bettarini S., Pasqualini E., Granata V., Busoni S., Messserini L. (2022). Gastroenteropancreatic neuroendocrine neoplasms (GEP-NENs): A radiomic model to predict tumor grade. Radiol. Med..

[77] Caruso D., Polici M., Rinzivillo M., Zerunian M., Nacci I., Marasco M., Magi L., Tarallo M., Gargiulo S., Iannicelli E. (2022). CT-based radiomics for prediction of therapeutic response to Everolimus in metastatic neuroendocrine tumors. Radiol. Med..

[78] Chiti G., Grazzini G., Cozzi D., Danti G., Matteuzzi B., Granata V., Pradella S., Recchia L., Brunese L., Miele V. (2021). Imaging of Pancreatic Neuroendocrine Neoplasms. Int. J. Environ. Res. Public Health.

[79] Fusco R., Setola S.V., Raiano N., Granata V., Cerciello V., Pecori B., Petrillo A. (2022). Analysis of a monocentric computed tomography dosimetric database using a radiation dose index monitoring software: Dose levels and alerts before and after the implementation of the adaptive statistical iterative reconstruction on CT images. Radiol. Med..

[80] Park S.H., Kim Y.S., Choi J. (2021). Dosimetric analysis of the effects of a temporary tissue expander on the radiotherapy technique. Radiol. Med..

[81] Bozkurt M., Eldem G., Bozbulut U.B., Bozkurt M.F., Kılıçkap S., Peynircioğlu B., Çil B., Lay Ergün E., Volkan-Salanci B. (2021). Factors affecting the response to Y-90 microsphere therapy in the cholangiocarcinoma patients. Radiol. Med..

[82] Horsley-Silva J.L., Rodriguez E.A., Franco D.L., Lindor K.D. (2017). An update on cancer risk and surveillance in primary sclerosing cholangitis. Liver Int..

[83] Mussa M., Martínez Pérez-Crespo P.M., Lopez-Cortes L.E., Retamar-Gentil P., Sousa-Dominguez A., Goikoetxea-Aguirre A.J., Reguera-Iglesias J.M., León Jiménez E., Fernández-Natal I., Armiñanzas-Castillo C. (2022). Risk Factors and Predictive Score for Bacteremic Biliary Tract Infections Due to Enterococcus faecalis and Enterococcus faecium: A Multicenter Cohort Study from the PROBAC Project. Microbiol. Spectr..

[84] Granata V., Coppola F., Grassi R., Fusco R., Tafuto S., Izzo F., Reginelli A., Maggialetti N., Buccicardi D., Frittoli B. (2021). Structured Reporting of Computed Tomography in the Staging of Neuroendocrine Neoplasms: A Delphi Consensus Proposal. Front. Endocrinol..

[85] Granata V., Simonetti I., Fusco R., Setola S.V., Izzo F., Scarpato L., Vanella V., Festino L., Simeone E., Ascierto P.A. (2022). Management of cutaneous melanoma: Radiologists challenging and risk assessment. Radiol. Med..

[86] Cirillo L., Rustici A., Toni F., Zoli M., Bartiromo F., Gramegna L.L., Cicala D., Tonon C., Caranci F., Lodi R. (2022). Vessel Wall MRI: Clinical implementation in cerebrovascular disorders-technical aspects. Radiol. Med..

[87] Granata V., Fusco R., De Muzio F., Cutolo C., Setola S.V., Dell’Aversana F., Grassi F., Belli A., Silvestro L., Ottaiano A. (2022). Radiomics and machine learning analysis based on magnetic resonance imaging in the assessment of liver mucinous colorectal metastases. Radiol. Med..

[88] Granata V., Caruso D., Grassi R., Cappabianca S., Reginelli A., Rizzati R., Masselli G., Golfieri R., Rengo M., Regge D. (2021). Structured Reporting of Rectal Cancer Staging and Restaging: A Consensus Proposal. Cancers.

[89] Granata V., De Muzio F., Cutolo C., Dell’Aversana F., Grassi F., Grassi R., Simonetti I., Bruno F., Palumbo P., Chiti G. (2022). Structured Reporting in Radiological Settings: Pitfalls and Perspectives. J. Pers. Med..

[90] Granata V., Pradella S., Cozzi D., Fusco R., Faggioni L., Coppola F., Grassi R., Maggialetti N., Buccicardi D., Lacasella G.V. (2021). Computed Tomography Structured Reporting in the Staging of Lymphoma: A Delphi Consensus Proposal. J. Clin. Med..

[91] Granata V., Faggioni L., Grassi R., Fusco R., Reginelli A., Rega D., Maggialetti N., Buccicardi D., Frittoli B., Rengo M. (2022). Structured reporting of computed tomography in the staging of colon cancer: A Delphi consensus proposal. Radiol. Med..

[92] Granata V., Morana G., D’Onofrio M., Fusco R., Coppola F., Grassi F., Cappabianca S., Reginelli A., Maggialetti N., Buccicardi D. (2021). Structured Reporting of Computed Tomography and Magnetic Resonance in the Staging of Pancreatic Adenocarcinoma: A Delphi Consensus Proposal. Diagnostics.

[93] Granata V., Grassi R., Miele V., Larici A.R., Sverzellati N., Cappabianca S., Brunese L., Maggialetti N., Borghesi A., Fusco R. (2021). Structured Reporting of Lung Cancer Staging: A Consensus Proposal. Diagnostics.

[94] Neri E., Granata V., Montemezzi S., Belli P., Bernardi D., Brancato B., Caumo F., Calabrese M., Coppola F., Cossu E. (2022). Structured reporting of X-ray mammography in the first diagnosis of breast cancer: A Delphi consensus proposal. Radiol. Med..

[95] Granata V., Fusco R., Catalano O., Filice S., Amato D.M., Nasti G., Avallone A., Izzo F., Petrillo A. (2015). Early Assessment of Colorectal Cancer Patients with Liver Metastases Treated with Antiangiogenic Drugs: The Role of Intravoxel Incoherent Motion in Diffusion-Weighted Imaging. PLoS ONE.

[96] Li N., Wakim J., Koethe Y., Huber T., Schenning R., Gade T.P., Hunt S.J., Park B.J. (2022). Multicenter assessment of augmented reality registration methods for image-guided interventions. Radiol. Med..

[97] Izzo F., Palaia R., Albino V., Amore A., di Giacomo R., Piccirillo M., Leongito M., Nasto A., Granata V., Petrillo A. (2015). Hepatocellular carcinoma and liver metastases: Clinical data on a new dual-lumen catheter kit for surgical sealant infusion to prevent perihepatic bleeding and dissemination of cancer cells following biopsy and loco-regional treatments. Infect. Agent. Cancer.

[98] Granata V., Fusco R., de Lutio di Castelguidone E., Avallone A., Palaia R., Delrio P., Tatangelo F., Botti G., Grassi R., Izzo F. (2019). Diagnostic performance of gadoxetic acid-enhanced liver MRI versus multidetector CT in the assessment of colorectal liver metastases compared to hepatic resection. BMC Gastroenterol..

[99] Fushimi Y., Yoshida K., Okawa M., Maki T., Nakajima S., Sakata A., Okuchi S., Hinoda T., Kanagaki M., Nakamoto Y. (2022). Vessel wall MR imaging in neuroradiology. Radiol. Med..

[100] Liu J., Wang C., Guo W., Zeng P., Liu Y., Lang N., Yuan H. (2021). A preliminary study using spinal MRI-based radiomics to predict high-risk cytogenetic abnormalities in multiple myeloma. Radiol. Med..

[101] Hussein M.A.M., Cafarelli F.P., Paparella M.T., Rennie W.J., Guglielmi G. (2021). Phosphaturic mesenchymal tumors: Radiological aspects and suggested imaging pathway. Radiol. Med..

[102] Pizzini F.B., Conti E., Bianchetti A., Splendiani A., Fusco D., Caranci F., Bozzao A., Landi F., Gandolfo N., Farina L. (2022). Radiological assessment of dementia: The Italian inter-society consensus for a practical and clinically oriented guide to image acquisition, evaluation, and reporting. Radiol. Med..

[103] Falcinelli L., Mendichi M., Chierchini S., Tenti M.V., Bellavita R., Saldi S., Ingrosso G., Reggioli V., Bini V., Aristei C. (2021). Pulmonary function in stereotactic body radiotherapy with helical tomotherapy for primary and metastatic lung lesions. Radiol. Med..

[104] Merlotti A., Bruni A., Borghetti P., Ramella S., Scotti V., Trovò M., Chiari R., Lohr F., Ricardi U., Bria E. (2021). Sequential chemo-hypofractionated RT versus concurrent standard CRT for locally advanced NSCLC: GRADE recommendation by the Italian Association of Radiotherapy and Clinical Oncology (AIRO). Radiol. Med..

[105] Barra S., Guarnieri A., di Monale E Bastia M.B., Marcenaro M., Tornari E., Belgioia L., Magrini S.M., Ricardi U., Corvò R. (2021). Short fractionation radiotherapy for early prostate cancer in the time of COVID-19: Long-term excellent outcomes from a multicenter Italian trial suggest a larger adoption in clinical practice. Radiol. Med..

[106] Cellini F., Di Franco R., Manfrida S., Borzillo V., Maranzano E., Pergolizzi S., Morganti A.G., Fusco V., Deodato F., Santarelli M. (2021). Palliative radiotherapy indications during the COVID-19 pandemic and in future complex logistic settings: The NORMALITY model. Radiol. Med..

[107] Lancellotta V., Del Regno L., Di Stefani A., Fionda B., Marazzi F., Rossi E., Balducci M., Pampena R., Morganti A.G., Mangoni M. (2022). The role of stereotactic radiotherapy in addition to immunotherapy in the management of melanoma brain metastases: Results of a systematic review. Radiol. Med..

[108] Laurelli G., Falcone F., Gallo M.S., Scala F., Losito S., Granata V., Cascella M., Greggi S. (2016). Long-Term Oncologic and Reproductive Outcomes in Young Women with Early Endometrial Cancer Conservatively Treated: A Prospective Study and Literature Update. Int. J. Gynecol Cancer.

[109] Granata V., Fusco R., Barretta M.L., Picone C., Avallone A., Belli A., Patrone R., Ferrante M., Cozzi D., Grassi R. (2021). Radiomics in hepatic metastasis by colorectal cancer. Infect Agent Cancer.

[110] Granata V., Fusco R., Costa M., Picone C., Cozzi D., Moroni C., La Casella G.V., Montanino A., Monti R., Mazzoni F. (2021). Preliminary Report on Computed Tomography Radiomics Features as Biomarkers to Immunotherapy Selection in Lung Adenocarcinoma Patients. Cancers.

[111] Tagliafico A.S., Campi C., Bianca B., Bortolotto C., Buccicardi D., Francesca C., Prost R., Rengo M., Faggioni L. (2022). Blockchain in radiology research and clinical practice: Current trends and future directions. Radiol. Med..

[112] Granata V., Fusco R., De Muzio F., Cutolo C., Setola S.V., Grassi R., Grassi F., Ottaiano A., Nasti G., Tatangelo F. (2022). Radiomics textural features by MR imaging to assess clinical outcomes following liver resection in colorectal liver metastases. Radiol. Med..

[113] Fusco R., Granata V., Sansone M., Rega D., Delrio P., Tatangelo F., Romano C., Avallone A., Pupo D., Giordano M. (2021). Validation of the standardized index of shape tool to analyze DCE-MRI data in the assessment of neo-adjuvant therapy in locally advanced rectal cancer. Radiol. Med..

[114] Renzulli M., Brandi N., Argalia G., Brocchi S., Farolfi A., Fanti S., Golfieri R. (2022). Morphological, dynamic and functional characteristics of liver pseudolesions and benign lesions. Radiol. Med..

[115] Chiloiro G., Cusumano D., de Franco P., Lenkowicz J., Boldrini L., Carano D., Barbaro B., Corvari B., Dinapoli N., Giraffa M. (2022). Does restaging MRI radiomics analysis improve pathological complete response prediction in rectal cancer patients? A prognostic model development. Radiol. Med..

[116] Granata V., Catalano O., Fusco R., Tatangelo F., Rega D., Nasti G., Avallone A., Piccirillo M., Izzo F., Petrillo A. (2015). The target sign in colorectal liver metastases: An atypical Gd-EOB-DTPA “uptake” on the hepatobiliary phase of MR imaging. Abdom. Imaging.

[117] Fusco R., Granata V., Rega D., Russo C., Pace U., Pecori B., Tatangelo F., Botti G., Izzo F., Cascella M. (2018). Morphological and functional features prognostic factor of magnetic resonance imaging in locally advanced rectal cancer. Acta Radiol..

[118] Fusco R., Petrillo M., Granata V., Filice S., Sansone M., Catalano O., Petrillo A. (2017). Magnetic resonance imaging evaluation in neoadjuvant therapy of locally advanced rectal cancer: A systematic review. Radiol. Oncol..

[119] Ledda R.E., Silva M., McMichael N., Sartorio C., Branchi C., Milanese G., Nayak S.M., Sverzellati N. (2022). The diagnostic value of grey-scale inversion technique in chest radiography. Radiol. Med..

[120] Kang Y.J., Cho J.H., Hwang S.H. (2022). Diagnostic value of various criteria for deep lobe involvement in radiologic studies with parotid mass: A systematic review and meta-analysis. Radiol. Med..

[121] Borgheresi A., De Muzio F., Agostini A., Ottaviani L., Bruno A., Granata V., Fusco R., Danti G., Flammia F., Grassi R. (2022). Lymph Nodes Evaluation in Rectal Cancer: Where Do We Stand and Future Perspective. J. Clin. Med..

[122] Fusco R., Sansone M., Granata V., Grimm R., Pace U., Delrio P., Tatangelo F., Botti G., Avallone A., Pecori B. (2019). Diffusion and perfusion MR parameters to assess preoperative short-course radiotherapy response in locally advanced rectal cancer: A comparative explorative study among Standardized Index of Shape by DCE-MRI, intravoxel incoherent motion- and diffusion kurtosis imaging-derived parameters. Abdom. Radiol..

[123] Scola E., Desideri I., Bianchi A., Gadda D., Busto G., Fiorenza A., Amadori T., Mancini S., Miele V., Fainardi E. (2022). Assessment of brain tumors by magnetic resonance dynamic susceptibility contrast perfusion-weighted imaging and computed tomography perfusion: A comparison study. Radiol. Med..

[124] Vicini S., Bortolotto C., Rengo M., Ballerini D., Bellini D., Carbone I., Preda L., Laghi A., Coppola F., Faggioni L. (2022). A narrative review on current imaging applications of artificial intelligence and radiomics in oncology: Focus on the three most common cancers. Radiol. Med..

[125] Granata V., Fusco R., Venanzio Setola S., Sassaroli C., De Franciscis S., Delrio P., Danti G., Grazzini G., Faggioni L., Gabelloni M. (2022). Radiological assessment of peritoneal carcinomatosis: A primer for resident. Eur. Rev. Med. Pharmacol. Sci..

[126] Petrick J.L., Yang B., Altekruse S.F., Van Dyke A.L., Koshiol J., Graubard B.I., McGlynn K.A. (2017). Risk factors for intrahepatic and extrahepatic cholangiocarcinoma in the United States: A population-based study in SEER-Medicare. PLoS ONE.

[127] Cardinale V., Semeraro R., Torrice A., Gatto M., Napoli C., Bragazzi M.C., Gentile R., Alvaro D. (2010). Intra-hepatic and extra-hepatic cholangiocarcinoma: New insight into epidemiology and risk factors. World J. Gastrointest. Oncol..

[128] Dil B.H., Cameron J.L., Reddy S., Lum Y., Lipsett P.A., Nathan H., Pawlik T.M., Choti M.A., Wolfgang C.L., Schulick R.D. (2008). Choledochal cyst disease in children and adults: A 30-year single-institution experience. J. Am. Coll. Surg..

[129] Funabiki T., Matsubara T., Miyakawa S., Ishihara S. (2009). Pancreaticobiliary maljunction and carcinogenesis to biliary and pancreatic malignancy. Langenbecks Arch. Surg..

[130] Ishibashi H., Shimada M., Kamisawa T., Fujii H., Hamada Y., Kubota M., Urushihara N., Endo I., Nio M., Taguchi T. (2017). Japanese clinical practice guidelines for congenital biliary dilatation. J. Hepatobiliary Pancreat Sci..

[131] Ouaïssi M., Kianmanesh R., Belghiti J., Ragot E., Mentha G., Adham M., Troisi R.I., Pruvot F.R., Dugué L., Paye F. (2015). Todani Type II Congenital Bile Duct Cyst: European Multicenter Study of the French Surgical Association and Literature Review. Ann. Surg..

[132] Moslim M.A., Takahashi H., Seifarth F.G., Walsh R.M., Morris-Stiff G. (2016). Choledochal Cyst Disease in a Western Center: A 30-Year Experience. J. Gastrointest. Surg..

[133] Nicholl M., Pitt H.A., Wolf P., Cooney J., Kalayoglu M., Shilyansky J., Rikkers L.F. (2004). Choledochal cysts in western adults: Complexities compared to children. J. Gastrointest. Surg..

[134] Todani T., Watanabe Y., Fujii M., Toki A., Uemara S., Koike Y., Inoue T. (1985). Carcinoma arising from the bile duct in choledochal cyst and anomalous arrangement of the pancreatobiliary ductal union (in Japanese). Tan to Sui (Biliary Tract Pancreas).

[135] Baison G.N., Bonds M.M., Helton W.S., Kozarek R.A. (2019). Choledochal cysts: Similarities and differences between Asian and Western countries. World J. Gastroenterol..

[136] Labib P.L., Goodchild G., Pereira S.P. (2019). Molecular Pathogenesis of Cholangiocarcinoma. BMC Cancer.

[137] Jang H.J., Kim T.K., Burns P.N., Wilson S.R. (2015). CEUS: An essential component in a multimodality approach to small nodules in patients at high-risk for hepatocellular carcinoma. Eur. J. Radiol..

[138] De Muzio F., Cutolo C., Dell’Aversana F., Grassi F., Ravo L., Ferrante M., Danti G., Flammia F., Simonetti I., Palumbo P. (2022). Complications after Thermal Ablation of Hepatocellular Carcinoma and Liver Metastases: Imaging Findings. Diagnostics.

[139] De Re V., Caggiari L., De Zorzi M., Repetto O., Zignego A.L., Izzo F., Tornesello M.L., Buonaguro F.M., Mangia A., Sansonno D. (2015). Genetic diversity of the KIR/HLA system and susceptibility to hepatitis C virus-related diseases. PLoS ONE.

[140] Granata V., Fusco R., Avallone A., Catalano O., Filice F., Leongito M., Palaia R., Izzo F., Petrillo A. (2017). Major and ancillary magnetic resonance features of LI-RADS to assess HCC: An overview and update. Infect Agent Cancer..

[141] Cutolo C., Dell’Aversana F., Fusco R., Grazzini G., Chiti G., Simonetti I., Bruno F., Palumbo P., Pierpaoli L., Valeri T. (2022). Combined Hepatocellular-Cholangiocarcinoma: What the Multidisciplinary Team Should Know. Diagnostics.

[142] Barile A. (2021). Some thoughts and greetings from the new Editor-in-Chief. Radiol. Med..

[143] Šubrt Z., Vošmik M., Whitley A., Oliverius M., Gürlich R. (2022). Intrahepatic cholangiocarcinoma: Risk factors affecting survival of operated patients. Rozhl. Chir..

[144] Izquierdo-Sanchez L., Lamarca A., La Casta A., Buettner S., Utpatel K., Klümpen H.J., Adeva J., Vogel A., Lleo A., Fabris L. (2022). Cholangiocarcinoma landscape in Europe: Diagnostic, prognostic and therapeutic insights from the ENSCCA Registry. J. Hepatol..

[145] Massarweh N.N., El-Serag H.B. (2017). Epidemiology of Hepatocellular Carcinoma and Intrahepatic Cholangiocarcinoma. Cancer Control.

[146] Palmer W.C., Patel T. (2012). Are common factors involved in the pathogenesis of primary liver cancers? A meta-analysis of risk factors for intrahepatic cholangiocarcinoma. J. Hepatol..

[147] Shi J., Zhu L., Liu S., Xie W.F. (2005). A meta-analysis of case-control studies on the combined effect of hepatitis B and C virus infec- tions in causing hepatocellular carcinoma in China. Br. J. Cancer..

[148] Peng N., Li L., Qin X., Guo Y., Peng T., Xiao K.-y., Chen X.-g., Yang Y.-F., Su Z., Chen B. (2011). Evaluation of risk factors and clinicopathologic features for intrahepatic cholangiocarcinoma in Southern China: A possible role of hepatitis B virus. Ann. Surg. Oncol..

[149] Sithithaworn P., Yongvanit P., Duenngai K., Kiatsopit N., Pairojkul C. (2014). Roles of liver fluke infection as risk factor for cholangiocarcinoma. J. Hepato-Biliary Pancreat Sci..

[150] Izzo F., Mason M.C., Silberfein E.J., Massarweh N.N., Hsu C., Tran Cao H.S., Palaia R., Piccirillo M., Belli A., Patrone R. (2022). Long-Term Survival and Curative-Intent Treatment in Hepatitis B or C Virus-Associated Hepatocellular Carcinoma Patients Diagnosed during Screening. Biology.

[151] Omata M., Cheng A.L., Kokudo N., Kudo M., Lee J.M., Jia J., Tateishi R., Han K.H., Chawla Y.K., Shiina S. (2017). Asia Pacific clinical practice guidelines on the management of hepatocellular carcinoma: A 2017 update. Hepatol. Int..

[152] European Association for the Study of the Liver (2018). EASL clinical practice guidelines: Management of hepatocellular carcinoma. J. Hepatol..

[153] Granata V., Fusco R., Setola S.V., Picone C., Vallone P., Belli A., Incollingo P., Albino V., Tatangelo F., Izzo F. (2019). Microvascular invasion and grading in hepatocellular carcinoma: Correlation with major and ancillary features according to LIRADS. Abdom. Radiol..

[154] De Muzio F., Grassi F., Dell’Aversana F., Fusco R., Danti G., Flammia F., Chiti G., Valeri T., Agostini A., Palumbo P. (2022). A Narrative Review on LI-RADS Algorithm in Liver Tumors: Prospects and Pitfalls. Diagnostics.

[155] Granata V., Fusco R., Venanzio Setola S., Sandomenico F., Luisa Barretta M., Belli A., Palaia R., Tatangelo F., Grassi R., Izzo F. (2020). Major and ancillary features according to LI-RADS in the assessment of combined hepatocellular-cholangiocarcinoma. Radiol. Oncol..

[156] Granata V., Grassi R., Fusco R., Setola S.V., Belli A., Piccirillo M., Pradella S., Giordano M., Cappabianca S., Brunese L. (2021). Abbreviated MRI Protocol for the Assessment of Ablated Area in HCC Patients. Int. J. Environ. Res. Public Health.

[157] Granata V., Fusco R., Avallone A., Filice F., Tatangelo F., Piccirillo M., Grassi R., Izzo F., Petrillo A. (2017). Critical analysis of the major and ancillary imaging features of LI-RADS on 127 proven HCCs evaluated with functional and morphological MRI: Lights and shadows. Oncotarget.

[158] Barabino M., Gurgitano M., Fochesato C., Angileri S.A., Franceschelli G., Santambrogio R., Mariani N.M., Opocher E., Carrafiello G. (2021). LI-RADS to categorize liver nodules in patients at risk of HCC: Tool or a gadget in daily practice?. Radiol. Med..

[159] Nakamura Y., Higaki T., Honda Y., Tatsugami F., Tani C., Fukumoto W., Narita K., Kondo S., Akagi M., Awai K. (2021). Advanced CT techniques for assessing hepatocellular carcinoma. Radiol. Med..

[160] Pignata S., Gallo C., Daniele B., Elba S., Giorgio A., Capuano G., Adinolfi L.E., De Sio I., Izzo F., Farinati F. (2006). Characteristics at presentation and outcome of hepatocellular carcinoma (HCC) in the elderly. A study of the Cancer of the Liver Italian Program (CLIP). Crit. Rev. Oncol. Hematol..

[161] Van der Pol C.B., McInnes M.D.F., Salameh J.P., Levis B., Chernyak V., Sirlin C.B., Bashir M.R., Allen B.C., Burke L.M.B., Choi J.Y. (2022). CT/MRI and CEUS LI-RADS Major Features Association with Hepatocellular Carcinoma: Individual Patient Data Meta-Analysis. Radiology.

[162] Tse J.R., Shen L., Bird K.N., Yoon L., Kamaya A. (2022). Outcomes of LI-RADS US-2 Subthreshold Observations Detected on Surveillance Ultrasound. AJR Am. J. Roentgenol..

[163] Morgan T.A., Maturen K.E., Dahiya N., Sun M.R.M., Kamaya A., American College of Radiology Ultrasound Liver Imaging and Reporting Data System (US LI-RADS) Working Group (2018). US LI-RADS: Ultrasound liver imaging reporting and data system for screening and surveillance of hepatocellular carcinoma. Abdom. Radiol..

[164] Rodgers S.K., Fetzer D.T., Gabriel H., Seow J.H., Choi H.H., Maturen K.E., Wasnik A.P., Morgan T.A., Dahiya N., O’Boyle M.K. (2019). Role of US LI-RADS in the LI-RADS Algorithm. Radiographics.

[165] Sato Y., Sasaki M., Harada K., Aishima S., Fukusato T., Ojima H., Kanai Y., Kage M., Nakanuma Y., Tsubouchi H. (2014). Pathological diagnosis of flat epithelial lesions of the biliary tract with emphasis on biliary intraepithelial neoplasia. J. Gastroenterol..

[166] Ainechi S., Lee H. (2016). Updates on Precancerous Lesions of the Biliary Tract: Biliary Precancerous Lesion. Arch. Pathol. Lab. Med..

[167] Busturk O., Aishima S., Esposito I., WHO Classification of Tumours Editorial Board (2019). Bilairy intraepithelial neoplasia. Digestive System Tumours.

[168] Wu T.T., Levy M., Correa A.M., Rosen C.B., Abraham S.C. (2009). Biliary intraepithelial neoplasia in patients without chronic biliary disease: Analysis of liver explants with alcoholic cirrhosis, hepatitis C infection, and noncirrhotic liver diseases. Cancer.

[169] Nakanuma Y., Uesaka K., Okamura Y., Terada T., Fukumura Y., Kakuda Y., Sugino T., Sato Y., Taek J.K., Park Y.N. (2021). Reappraisal of pathological features of intraductal papillary neoplasm of bile duct with respect to the type 1 and 2 subclassifications. Hum. Pathol..

[170] Nakanuma Y., Uesaka K., Kakuda Y., Sugino T., Kubota K., Furukawa T., Fukumura Y., Isayama H., Terada T. (2020). Intraductal Papillary Neoplasm of Bile Duct: Updated Clinicopathological Characteristics and Molecular and Genetic Alterations. J. Clin. Med..

[171] Nakanuma Y., Busturk O., Esposito I., Klimstra D.S., Komuta M., Zen Y., WHO Classification of Tumours Editorial Board (2019). Intraductal Papillary Neoplasm of Bile Duct. Digestive System Tumours.

[172] Aoki Y., Mizuma M., Hata T., Aoki T., Omori Y., Ono Y., Mizukami Y., Unno M., Furukawa T. (2020). Intraductal papillary neoplasms of the bile duct consist of two distinct types specifically associated with clinicopathological features and molecular phenotypes. J. Pathol..

[173] Nakanuma Y., Jang K.T., Fukushima N., Furukawa T., Hong S.M., Kim H., Lee K.B., Zen Y., Jang J.Y., Kubota K. (2018). A statement by the Japan-Korea expert pathologists for future clinicopathological and molecular analyses toward consensus building of intraductal papillary neoplasm of the bile duct through several opinions at the present stage. J. Hepatobiliary Pancreat Sci..

[174] Nakanuma Y., Uesaka K., Miyayama S., Yamaguchi H., Ohtsuka M. (2017). Intraductal neoplasms of the bile duct. A new challenge to biliary tract tumor pathology. Histol. Histopathol..

[175] Lendvai G., Szekerczés T., Illyés I., Dóra R., Kontsek E., Gógl A., Kiss A., Werling K., Kovalszky I., Schaff Z. (2020). Cholangiocarcinoma: Classification, Histopathology and Molecular Carcinogenesis. Pathol. Oncol. Res..

[176] Akita M., Hong S.M., Sung Y.N., Kim M.J., Ajiki T., Fukumoto T., Itoh T., Zen Y. (2020). Biliary intraductal tubule-forming neoplasm: A whole exome sequencing study of MUC5AC-positive and -negative cases. Histopathology.

[177] Van Treeck B.J., Lotfalla M., Czeczok T.W., Mounajjed T., Moreira R.K., Allende D.S., Reid M.D., Naini B.V., Westerhoff M., Adsay N.V. (2020). Molecular and Immunohistochemical Analysis of Mucinous Cystic Neoplasm of the Liver. Am. J. Clin. Pathol..

[178] Zen Y., Pedica F., Patcha V.R., Capelli P., Zamboni G., Casaril A., Quaglia A., Nakanuma Y., Heaton N., Portmann B. (2011). Mucinous cystic neoplasms of the liver: A clinicopathological study and comparison with intraductal papillary neoplasms of the bile duct. Mod. Pathol..

[179] Busturk O., Nakanuma Y., Aishima A., Esposito I., WHO Classification of Tumours Editorial Board (2019). Mucinous cystic neoplasms of the liver and biliary systtem. Digestive System Tumours.

[180] Granata V., Fusco R.M., Catalano O., Filice S., Avallone A., Piccirillo M., Leongito M., Palaia R., Grassi R., Izzo F. (2017). Uncommon neoplasms of the biliary tract: Radiological findings. Br. J. Radiol..

[181] Petralia G., Zugni F., Summers P.E., Colombo A., Pricolo P., Grazioli L., Colagrande S., Giovagnoni A., Padhani A.R., Italian Working Group on Magnetic Resonance (2021). Whole-body magnetic resonance imaging (WB-MRI) for cancer screening: Recommendations for use. Radiol. Med..

[182] Bianchi A., Mazzoni L.N., Busoni S., Pinna N., Albanesi M., Cavigli E., Cozzi D., Poggesi A., Miele V., Fainardi E. (2021). Assessment of cerebrovascular disease with computed tomography in COVID-19 patients: Correlation of a novel specific visual score with increased mortality risk. Radiol. Med..

[183] Cartocci G., Colaiacomo M.C., Lanciotti S., Andreoli C., De Cicco M.L., Brachetti G., Pugliese S., Capoccia L., Tortora A., Scala A. (2021). Correction to: Chest CT for early detection and management of coronavirus disease (COVID-19): A report of 314 patients admitted to Emergency Department with suspected pneumonia. Radiol. Med..

[184] Sansone M., Marrone S., Di Salvio G., Belfiore M.P., Gatta G., Fusco R., Vanore L., Zuiani C., Grassi F., Vietri M.T. (2022). Comparison between two packages for pectoral muscle removal on mammographic images. Radiol. Med..

[185] Han D., Yu N., Yu Y., He T., Duan X. (2022). Performance of CT radiomics in predicting the overall survival of patients with stage III clear cell renal carcinoma after radical nephrectomy. Radiol. Med..

[186] Masci G.M., Ciccarelli F., Mattei F.I., Grasso D., Accarpio F., Catalano C., Laghi A., Sammartino P., Iafrate F. (2022). Role of CT texture analysis for predicting peritoneal metastases in patients with gastric cancer. Radiol. Med..

[187] Fusco R., Granata V., Mazzei M.A., Meglio N.D., Roscio D.D., Moroni C., Monti R., Cappabianca C., Picone C., Neri E. (2021). Quantitative imaging decision support (QIDSTM) tool consistency evaluation and radiomic analysis by means of 594 metrics in lung carcinoma on chest CT scan. Cancer Control..

[188] Zerunian M., Pucciarelli F., Caruso D., Polici M., Masci B., Guido G., De Santis D., Polverari D., Principessa D., Benvenga A. (2022). Artificial intelligence based image quality enhancement in liver MRI: A quantitative and qualitative evaluation. Radiol. Med..

[189] Petrillo A., Fusco R., Petrillo M., Granata V., Delrio P., Bianco F., Pecori B., Botti G., Tatangelo F., Caracò C. (2017). Standardized Index of Shape (DCE-MRI) and Standardized Uptake Value (PET/CT): Two quantitative approaches to discriminate chemo-radiotherapy locally advanced rectal cancer responders under a functional profile. Oncotarget.

[190] Masci G.M., Iafrate F., Ciccarelli F., Pambianchi G., Panebianco V., Pasculli P., Ciardi M.R., Mastroianni C.M., Ricci P., Catalano C. (2021). Tocilizumab effects in COVID-19 pneumonia: Role of CT texture analysis in quantitative assessment of response to therapy. Radiol. Med..

[191] Francolini G., Desideri I., Stocchi G., Ciccone L.P., Salvestrini V., Garlatti P., Aquilano M., Greto D., Bonomo P., Meattini I. (2021). Impact of COVID-19 on workload burden of a complex radiotherapy facility. Radiol. Med..

[192] Bruno F., Granata V., Cobianchi Bellisari F., Sgalambro F., Tommasino E., Palumbo P., Arrigoni F., Cozzi D., Grassi F., Brunese M.C. (2022). Advanced Magnetic Resonance Imaging (MRI) Techniques: Technical Principles and Applications in Nanomedicine. Cancers.

[193] De Robertis R., Geraci L., Tomaiuolo L., Bortoli L., Beleù A., Malleo G., D’Onofrio M. (2022). Liver metastases in pancreatic ductal adenocarcinoma: A predictive model based on CT texture analysis. Radiol. Med..

[194] Gurgitano M., Angileri S.A., Rodà G.M., Liguori A., Pandolfi M., Ierardi A.M., Wood B.J., Carrafiello G. (2021). Interventional Radiology ex-machina: Impact of Artificial Intelligence on practice. Radiol. Med..

[195] Assadsangabi R., Babaei R., Songco C., Ivanovic V., Bobinski M., Chen Y.J., Nabavizadeh S.A. (2021). Multimodality oncologic evaluation of superficial neck and facial lymph nodes. Radiol. Med..

[196] Giurazza F., Contegiacomo A., Calandri M., Mosconi C., Modestino F., Corvino F., Scrofani A.R., Marra P., Coniglio G., Failla G. (2021). IVC filter retrieval: A multicenter proposal of two score systems to predict application of complex technique and procedural outcome. Radiol. Med..

[197] Granata V., Fusco R., Bicchierai G., Cozzi D., Grazzini G., Danti G., De Muzio F., Maggialetti N., Smorchkova O., D’Elia M. (2021). Diagnostic protocols in oncology: Workup and treatment planning: Part 1: The optimitation of CT protocol. Eur. Rev. Med. Pharmacol. Sci..

[198] Granata V., Bicchierai G., Fusco R., Cozzi D., Grazzini G., Danti G., De Muzio F., Maggialetti N., Smorchkova O., D’Elia M. (2021). Diagnostic protocols in oncology: Workup and treatment planning. Part 2: Abbreviated MR protocol. Eur. Rev. Med. Pharmacol. Sci..

[199] Granata V., Fusco R., Belli A., Danti G., Bicci E., Cutolo C., Petrillo A., Izzo F. (2022). Diffusion weighted imaging and diffusion kurtosis imaging in abdominal oncological setting: Why and when. Infect. Agent Cancer.

[200] Izzo F., Granata V., Grassi R., Fusco R., Palaia R., Delrio P., Carrafiello G., Azoulay D., Petrillo A., Curley S.A. (2019). Radiofrequency Ablation and Microwave Ablation in Liver Tumors: An Update. Oncologist.

[201] Granata V., Grassi R., Fusco R., Galdiero R., Setola S.V., Palaia R., Belli A., Silvestro L., Cozzi D., Brunese L. (2021). Pancreatic cancer detection and characterization: State of the art and radiomics. Eur. Rev. Med. Pharmacol. Sci..

[202] Granata V., Fusco R., Risi C., Ottaiano A., Avallone A., De Stefano A., Grimm R., Grassi R., Brunese L., Izzo F. (2020). Diffusion-Weighted MRI and Diffusion Kurtosis Imaging to Detect RAS Mutation in Colorectal Liver Metastasis. Cancers.

[203] Perillo T., Paolella C., Perrotta G., Serino A., Caranci F., Manto A. (2022). Reversible cerebral vasoconstriction syndrome: Review of neuroimaging findings. Radiol. Med..

[204] Petrillo A., Fusco R., Granata V., Filice S., Sansone M., Rega D., Delrio P., Bianco F., Romano G.M., Tatangelo F. (2018). Assessing response to neo-adjuvant therapy in locally advanced rectal cancer using Intra-voxel Incoherent Motion modelling by DWI data and Standardized Index of Shape from DCE-MRI. Ther. Adv. Med. Oncol..

[205] De Felice F., Boldrini L., Greco C., Nardone V., Salvestrini V., Desideri I. (2021). ESTRO vision 2030: The young Italian Association of Radiotherapy and Clinical Oncology (yAIRO) commitment statement. Radiol. Med..

[206] Rocca A., Brunese M.C., Santone A., Avella P., Bianco P., Scacchi A., Scaglione M., Bellifemine F., Danzi R., Varriano G. (2021). Early Diagnosis of Liver Metastases from Colorectal Cancer through CT Radiomics and Formal Methods: A Pilot Study. J. Clin. Med..

[207] Wei J., Cheng J., Gu D., Chai F., Hong N., Wang Y., Tian J. (2021). Deep learning-based radiomics predicts response to chemotherapy in colorectal liver metastases. Med. Phys..

[208] Saini A., Breen I., Pershad Y., Naidu S., Knuttinen M.G., Alzubaidi S., Sheth R., Albadawi H., Kuo M., Oklu R. (2018). Radiogenomics and Radiomics in Liver Cancers. Diagnostics.

[209] Petrillo A., Fusco R., Di Bernardo E., Petrosino T., Barretta M.L., Porto A., Granata V., Di Bonito M., Fanizzi A., Massafra R. (2022). Prediction of Breast Cancer Histological Outcome by Radiomics and Artificial Intelligence Analysis in Contrast-Enhanced Mammography. Cancers.

[210] Granata V., Fusco R., De Muzio F., Cutolo C., Setola S.V., Dell’Aversana F., Belli A., Romano C., Ottaiano A., Nasti G. (2022). Magnetic Resonance Features of Liver Mucinous Colorectal Metastases: What the Radiologist Should Know. J. Clin. Med..

[211] Wang Y., Ma L.Y., Yin X.P., Gao B.L. (2022). Radiomics and Radiogenomics in Evaluation of Colorectal Cancer Liver Metastasis. Front. Oncol..

[212] Costa G., Cavinato L., Masci C., Fiz F., Sollini M., Politi L.S., Chiti A., Balzarini L., Aghemo A., di Tommaso L. (2021). Virtual Biopsy for Diagnosis of Chemotherapy-Associated Liver Injuries and Steatohepatitis: A Combined Radiomic and Clinical Model in Patients with Colorectal Liver Metastases. Cancers.

[213] Donato H., França M., Candelária I., Caseiro-Alves F. (2017). Liver MRI: From basic protocol to advanced techniques. Eur. J. Radiol..

[214] Ligero M., Jordi-Ollero O., Bernatowicz K., Garcia-Ruiz A., Delgado-Muñoz E., Leiva D., Mast R., Suarez C., Sala-Llonch R., Calvo N. (2021). Minimizing acquisition-related radiomics variability by image resampling and batch effect correction to allow for large-scale data analysis. Eur. Radiol..

[215] Granata V., Fusco R., Setola S.V., Galdiero R., Picone C., Izzo F., D’Aniello R., Miele V., Grassi R., Grassi R. (2021). Lymphadenopathy after BNT162b2 Covid-19 Vaccine: Preliminary Ultrasound Findings. Biology.

[216] Scapicchio C., Gabelloni M., Barucci A., Cioni D., Saba L., Neri E. (2021). A deep look into radiomics. Radiol. Med..

[217] Morin O., Vallières M., Jochems A., Woodruff H.C., Valdes G., Braunstein S.E., Wildberger J.E., Villanueva-Meyer J.E., Kearney V., Yom S.S. (2018). A Deep Look Into the Future of Quantitative Imaging in Oncology: A Statement of Working Principles and Proposal for Change. Int. J. Radiat. Oncol. Biol. Phys..

[218] Cellina M., Pirovano M., Ciocca M., Gibelli D., Floridi C., Oliva G. (2021). Radiomic analysis of the optic nerve at the first episode of acute optic neuritis: An indicator of optic nerve pathology and a predictor of visual recovery?. Radiol. Med..

[219] Santone A., Brunese M.C., Donnarumma F., Guerriero P., Mercaldo F., Reginelli A., Miele V., Giovagnoni A., Brunese L. (2021). Radiomic features for prostate cancer grade detection through formal verification. Radiol. Med..

[220] Agazzi G.M., Ravanelli M., Roca E., Medicina D., Balzarini P., Pessina C., Vermi W., Berruti A., Maroldi R., Farina D. (2021). CT texture analysis for prediction of EGFR mutational status and ALK rearrangement in patients with non-small cell lung cancer. Radiol. Med..

[221] Benedetti G., Mori M., Panzeri M.M., Barbera M., Palumbo D., Sini C., Muffatti F., Andreasi V., Steidler S., Doglioni C. (2021). CT-derived radiomic features to discriminate histologic characteristics of pancreatic neuroendocrine tumors. Radiol. Med..

[222] Calloni S.F., Panni P., Calabrese F., Del Poggio A., Roveri L., Squarza S., Pero G.C., Paolucci A., Filippi M., Falini A. (2022). Cerebral hyperdensity on CT imaging (CTHD) post-reperfusion treatment in patients with acute cerebral stroke: Understanding its clinical meaning. Radiol. Med..

[223] Granata V., Fusco R., Avallone A., De Stefano A., Ottaiano A., Sbordone C., Brunese L., Izzo F., Petrillo A. (2021). Radiomics-Derived Data by Contrast Enhanced Magnetic Resonance in RAS Mutations Detection in Colorectal Liver Metastases. Cancers.

[224] Halefoglu A.M., Ozagari A.A. (2021). Tumor grade estımatıon of clear cell and papıllary renal cell carcınomas usıng contrast-enhanced MDCT and FSE T2 weıghted MR ımagıng: Radıology-pathology correlatıon. Radiol. Med..

[225] Granata V., Fusco R., Setola S.V., Simonetti I., Cozzi D., Grazzini G., Grassi F., Belli A., Miele V., Izzo F. (2022). An update on radiomics techniques in primary liver cancers. Infect Agent Cancer..

[226] Chen J., Zhang C., Traverso A., Zhovannik I., Dekker A., Wee L., Bermejo I. (2021). Generative models improve radiomics reproducibility in low dose CTs: A simulation study. Phys. Med. Biol..

[227] Arrigoni F., Mazzoleni M.G., Calvisi V., Masciocchi C. (2022). In-Office Needle Arthroscopy (IONA): May a traditionally orthopedic procedure enter the portfolio of interventional radiology?. Radiol. Med..

[228] Granata V., Fusco R., Sansone M., Grassi R., Maio F., Palaia R., Tatangelo F., Botti G., Grimm R., Curley S. (2020). Magnetic resonance imaging in the assessment of pancreatic cancer with quantitative parameter extraction by means of dynamic contrast-enhanced magnetic resonance imaging, diffusion kurtosis imaging and intravoxel incoherent motion diffusion-weighted imaging. Therap. Adv. Gastroenterol..

[229] Granata V., Palaia R., Albino V., Piccirillo M., Venanzio Setola S., Petrillo A., Izzo F. (2020). Electrochemotherapy of cholangiocellular carcinoma at hepatic hilum: A case report. Eur. Rev. Med. Pharmacol. Sci..

[230] Grassi R., Cappabianca S., Urraro F., Feragalli B., Montanelli A., Patelli G., Granata V., Giacobbe G., Russo G.M., Grillo A. (2020). Chest CT Computerized Aided Quantification of PNEUMONIA Lesions in COVID-19 Infection: A Comparison among Three Commercial Software. Int. J. Environ. Res. Public Health.

[231] Fusco R., Grassi R., Granata V., Setola S.V., Grassi F., Cozzi D., Pecori B., Izzo F., Petrillo A. (2021). Artificial Intelligence and COVID-19 Using Chest CT Scan and Chest X-ray Images: Machine Learning and Deep Learning Approaches for Diagnosis and Treatment. J. Pers. Med..

[232] Ozel M., Aslan A., Araç S. (2021). Use of the COVID-19 Reporting and Data System (CO-RADS) classification and chest computed tomography involvement score (CT-IS) in COVID-19 pneumonia. Radiol. Med..

[233] Ippolito D., Giandola T., Maino C., Pecorelli A., Capodaglio C., Ragusi M., Porta M., Gandola D., Masetto A., Drago S. (2021). Acute pulmonary embolism in hospitalized patients with SARS-CoV-2-related pneumonia: Multicentric experience from Italian endemic area. Radiol. Med..

[234] Moroni C., Cozzi D., Albanesi M., Cavigli E., Bindi A., Luvarà S., Busoni S., Mazzoni L.N., Grifoni S., Nazerian P. (2021). Chest X-ray in the emergency department during COVID-19 pandemic descending phase in Italy: Correlation with patients’ outcome. Radiol. Med..

[235] Cereser L., Girometti R., Da Re J., Marchesini F., Como G., Zuiani C. (2021). Inter-reader agreement of high-resolution computed tomography findings in patients with COVID-19 pneumonia: A multi-reader study. Radiol. Med..

[236] Rawashdeh M.A., Saade C. (2021). Radiation dose reduction considerations and imaging patterns of ground glass opacities in coronavirus: Risk of over exposure in computed tomography. Radiol. Med..

[237] Granata V., Ianniello S., Fusco R., Urraro F., Pupo D., Magliocchetti S., Albarello F., Campioni P., Cristofaro M., Di Stefano F. (2021). Quantitative Analysis of Residual COVID-19 Lung CT Features: Consistency among Two Commercial Software. J. Pers. Med..

[238] Fusco R., Granata V., Petrillo A. (2020). Introduction to Special Issue of Radiology and Imaging of Cancer. Cancers.

[239] Fusco R., Sansone M., Filice S., Granata V., Catalano O., Amato D.M., Di Bonito M., D’Aiuto M., Capasso I., Rinaldo M. (2015). Integration of DCE-MRI and DW-MRI Quantitative Parameters for Breast Lesion Classification. Biomed. Res. Int..

[240] Nakamoto T., Haga A., Takahashi W. (2018). An Introduction to Radiomics: Toward a New Era of Precision Medicine. Igaku Butsuri..

[241] Vuong D., Tanadini-Lang S., Wu Z., Marks R., Unkelbach J., Hillinger S., Eboulet E.I., Thierstein S., Peters S., Pless M. (2020). Radiomics Feature Activation Maps as a New Tool for Signature Interpretability. Front. Oncol..

[242] Yip S.S., Aerts H.J. (2016). Applications and limitations of radiomics. Phys. Med. Biol..

[243] Granata V., Fusco R., De Muzio F., Cutolo C., Setola S.V., Simonetti I., Dell’Aversana F., Grassi F., Bruno F., Belli A. (2022). Complications Risk Assessment and Imaging Findings of Thermal Ablation Treatment in Liver Cancers: What the Radiologist Should Expect. J. Clin. Med..

[244] Wilson R., Devaraj A. (2017). Radiomics of pulmonary nodules and lung cancer. Transl. Lung Cancer Res..

[245] Binczyk F., Prazuch W., Bozek P., Polanska J. (2021). Radiomics and artificial intelligence in lung cancer screening. Transl. Lung Cancer Res..

[246] Beig N., Bera K., Tiwari P. (2021). Introduction to radiomics and radiogenomics in neuro-oncology: Implications and challenges. Neurooncol. Adv..

[247] Barile A., Lanni G., Conti L., Mariani S., Calvisi V., Castagna A., Rossi F., Masciocchi C. (2013). Lesions of the biceps pulley as cause of anterosuperior impingement of the shoulder in the athlete: Potentials and limits of MR arthrography compared with arthroscopy. Radiol. Med..

[248] Masciocchi C., Lanni G., Conti L., Conchiglia A., Fascetti E., Flamini S., Coletti G., Barile A. (2012). Soft-tissue inflammatory myofibroblastic tumors (IMTs) of the limbs: Potential and limits of diagnostic imaging. Skeletal. Radiol..

[249] Chen Q., Zhang L., Liu S., You J., Chen L., Jin Z., Zhang S., Zhang B. (2022). Radiomics in precision medicine for gastric cancer: Opportunities and challenges. Eur. Radiol..

[250] Shi Z., Traverso A., van Soest J., Dekker A., Wee L. (2019). Technical Note: Ontology-guided radiomics analysis workflow (O-RAW). Med. Phys..

[251] Granata V., Fusco R., Setola S.V., De Muzio F., Dell’ Aversana F., Cutolo C., Faggioni L., Miele V., Izzo F., Petrillo A. (2022). CT-Based Radiomics Analysis to Predict Histopathological Outcomes Following Liver Resection in Colorectal Liver Metastases. Cancers.

[252] Sun J., Li H., Gao J., Li J., Li M., Zhou Z., Peng Y. (2021). Performance evaluation of a deep learning image reconstruction (DLIR) algorithm in “double low” chest CTA in children: A feasibility study. Radiol. Med..

[253] Granata V., Fusco R., De Muzio F., Cutolo C., Setola S.V., Dell’Aversana F., Ottaiano A., Nasti G., Grassi R., Pilone V. (2022). EOB-MR Based Radiomics Analysis to Assess Clinical Outcomes following Liver Resection in Colorectal Liver Metastases. Cancers.

[254] Granata V., Fusco R., De Muzio F., Cutolo C., Setola S.V., Dell’ Aversana F., Ottaiano A., Avallone A., Nasti G., Grassi F. (2022). Contrast MR-Based Radiomics and Machine Learning Analysis to Assess Clinical Outcomes following Liver Resection in Colorectal Liver Metastases: A Preliminary Study. Cancers.

[255] Granata V., Fusco R., De Muzio F., Cutolo C., Mattace Raso M., Gabelloni M., Avallone A., Ottaiano A., Tatangelo F., Brunese M.C. (2022). Radiomics and Machine Learning Analysis Based on Magnetic Resonance Imaging in the Assessment of Colorectal Liver Metastases Growth Pattern. Diagnostics.

[256] Chianca V., Albano D., Messina C., Vincenzo G., Rizzo S., Del Grande F., Sconfienza L.M. (2021). An update in musculoskeletal tumors: From quantitative imaging to radiomics. Radiol. Med..

[257] Qin H., Que Q., Lin P., Li X., Wang X.R., He Y., Chen J.Q., Yang H. (2021). Magnetic resonance imaging (MRI) radiomics of papillary thyroid cancer (PTC): A comparison of predictive performance of multiple classifiers modeling to identify cervical lymph node metastases before surgery. Radiol. Med..

[258] Fusco R., Di Bernardo E., Piccirillo A., Rubulotta M.R., Petrosino T., Barretta M.L., Mattace Raso M., Vallone P., Raiano C., Di Giacomo R. (2022). Radiomic and Artificial Intelligence Analysis with Textural Metrics Extracted by Contrast-Enhanced Mammography and Dynamic Contrast Magnetic Resonance Imaging to Detect Breast Malignant Lesions. Curr. Oncol..

[259] Brunese L., Brunese M.C., Carbone M., Ciccone V., Mercaldo F., Santone A. (2022). Automatic PI-RADS assignment by means of formal methods. Radiol. Med..

[260] Bellardita L., Colciago R.R., Frasca S., De Santis M.C., Gay S., Palorini F., La Rocca E., Valdagni R., Rancati T., Lozza L. (2021). Breast cancer patient perspective on opportunities and challenges of a genetic test aimed to predict radio-induced side effects before treatment: Analysis of the Italian branch of the REQUITE project. Radiol. Med..

[261] Caruso D., Pucciarelli F., Zerunian M., Ganeshan B., De Santis D., Polici M., Rucci C., Polidori T., Guido G., Bracci B. (2021). Chest CT texture-based radiomics analysis in differentiating COVID-19 from other interstitial pneumonia. Radiol. Med..

[262] Matsoukas S., Scaggiante J., Schuldt B.R., Smith C.J., Chennareddy S., Kalagara R., Majidi S., Bederson J.B., Fifi J.T., Mocco J. (2022). Accuracy of artificial intelligence for the detection of intracranial hemorrhage and chronic cerebral microbleeds: A systematic review and pooled analysis. Radiol. Med..

[263] Karmazanovsky G., Gruzdev I., Tikhonova V., Kondratyev E., Revishvili A. (2021). Computed tomography-based radiomics approach in pancreatic tumors characterization. Radiol. Med..

[264] Satake H., Ishigaki S., Ito R., Naganawa S. (2022). Radiomics in breast MRI: Current progress toward clinical application in the era of artificial intelligence. Radiol. Med..

[265] Gregucci F., Fiorentino A., Mazzola R., Ricchetti F., Bonaparte I., Surgo A., Figlia V., Carbonara R., Caliandro M., Ciliberti M.P. (2022). Radiomic analysis to predict local response in locally advanced pancreatic cancer treated with stereotactic body radiation therapy. Radiol. Med..

[266] Ji G.W., Wang K., Xia Y.X., Li X.C., Wang X.H. (2020). Application and challenge of radiomics technique in the era of precision medicine for hepatobiliary disease. Zhonghua Wai Ke Za Zhi.

[267] Wu J., Tha K.K., Xing L., Li R. (2018). Radiomics and radiogenomics for precision radiotherapy. J. Radiat Res..

[268] Rizzo S., Botta F., Raimondi S., Origgi D., Fanciullo C., Morganti A.G., Bellomi M. (2018). Radiomics: The facts and the challenges of image analysis. Eur. Radiol. Exp..

[269] Orlhac F., Nioche C., Klyuzhin I., Rahmim A., Buvat I. (2021). Radiomics in PET Imaging: A Practical Guide for Newcomers. PET Clin..

[270] Avanzo M., Stancanello J., El Naqa I. (2017). Beyond imaging: The promise of radiomics. Phys. Med..

[271] Lambin P., Rios-Velazquez E., Leijenaar R., Carvalho S., van Stiphout R.G., Granton P., Zegers C.M., Gillies R., Boellard R., Dekker A and Aerts H.J. (2012). Radiomics: Extracting more information from medical images using advanced feature analysis. Eur. J. Cancer.

[272] Gebauer L., Moltz J.H., Mühlberg A., Holch J.W., Huber T., Enke J., Jäger N., Haas M., Kruger S., Boeck S. (2021). Quantitative Imaging Biomarkers of the Whole Liver Tumor Burden Improve Survival Prediction in Metastatic Pancreatic Cancer. Cancers.

[273] Euler A., Laqua F.C., Cester D., Lohaus N., Sartoretti T., Pinto Dos Santos D., Alkadhi H., Baessler B. (2021). Virtual Monoenergetic Images of Dual-Energy CT-Impact on Repeatability, Reproducibility, and Classification in Radiomics. Cancers.

[274] Kelahan L.C., Kim D., Soliman M., Avery R.J., Savas H., Agrawal R., Magnetta M., Liu B.P., Velichko Y.S. (2022). Role of hepatic metastatic lesion size on inter-reader reproducibility of CT-based radiomics features. Eur. Radiol..

[275] Bracco S., Zanoni M., Casseri T., Castellano D., Cioni S., Vallone I.M., Gennari P., Mazzei M.A., Romano D.G., Piano M. (2021). Endovascular treatment of acute ischemic stroke due to tandem lesions of the anterior cerebral circulation: A multicentric Italian observational study. Radiol. Med..

[276] Michallek F., Genske U., Niehues S.M., Hamm B., Jahnke P. (2022). Deep learning reconstruction improves radiomics feature stability and discriminative power in abdominal CT imaging: A phantom study. Eur. Radiol..

[277] Fusco R., Sansone M., Granata V., Setola S.V., Petrillo A. (2017). A systematic review on multiparametric MR imaging in prostate cancer detection. Infect Agent Cancer..

[278] Cappabianca S., Granata V., Di Grezia G., Mandato Y., Reginelli A., Di Mizio V., Grassi R., Rotondo A. (2011). The role of nasoenteric intubation in the MR study of patients with Crohn’s disease: Our experience and literature review. Radiol. Med..

[279] De Filippo M., Puglisi S., D’Amuri F., Gentili F., Paladini I., Carrafiello G., Maestroni U., Del Rio P., Ziglioli F., Pagnini F. (2021). CT-guided percutaneous drainage of abdominopelvic collections: A pictorial essay. Radiol. Med..

[280] Pecoraro M., Cipollari S., Marchitelli L., Messina E., Del Monte M., Galea N., Ciardi M.R., Francone M., Catalano C., Panebianco V. (2021). Cross-sectional analysis of follow-up chest MRI and chest CT scans in patients previously affected by COVID-19. Radiol. Med..

[281] Gabelloni M., Faggioni L., Cioni D., Mendola V., Falaschi Z., Coppola S., Corradi F., Isirdi A., Brandi N., Coppola F. (2022). Extracorporeal membrane oxygenation (ECMO) in COVID-19 patients: A pocket guide for radiologists. Radiol. Med..

[282] Mayerhoefer M.E., Materka A., Langs G., Häggström I., Szczypiński P., Gibbs P., Cook G. (2020). Introduction to Radiomics. J. Nucl. Med..

[283] Zhang Z., Shen L., Wang Y., Wang J., Zhang H., Xia F., Wan J., Zhang Z. (2021). MRI Radiomics Signature as a Potential Biomarker for Predicting KRAS Status in Locally Advanced Rectal Cancer Patients. Front Oncol..

[284] Yang L., Dong D., Fang M., Zhu Y., Zang Y., Liu Z., Zhang H., Ying J., Zhao X., Tian J. (2018). Can Ct-Based Radiomics Signature Predict Kras/Nras/Braf Mutations in Colorectal Cancer?. Eur. Radiol..

[285] Li Wen Y., Leech M. (2020). Review of the Role of Radiomics in Tumour Risk Classification and Prognosis of Cancer. Anticancer Res..

[286] Agostini A., Borgheresi A., Carotti M., Ottaviani L., Badaloni M., Floridi C., Giovagnoni A. (2021). Third-generation iterative reconstruction on a dual-source, high-pitch, low-dose chest CT protocol with tin filter for spectral shaping at 100 kV: A study on a small series of COVID-19 patients. Radiol. Med..

[287] Palmisano A., Scotti G.M., Ippolito D., Morelli M.J., Vignale D., Gandola D., Sironi S., De Cobelli F., Ferrante L., Spessot M. (2021). Chest CT in the emergency department for suspected COVID-19 pneumonia. Radiol. Med..

[288] Lombardi A.F., Afsahi A.M., Gupta A., Gholamrezanezhad A. (2021). Severe acute respiratory syndrome (SARS), Middle East respiratory syndrome (MERS), influenza, and COVID-19, beyond the lungs: A review article. Radiol. Med..

[289] Golia Pernicka J.S., Gagniere J., Chakraborty J., Yamashita R., Nardo L., Creasy J.M., Petkovska I., Do R.R., Bates D.D., Paroder V. (2019). Radiomics-Based Prediction of Microsatellite Instability in Colorectal Cancer at Initial Computed Tomography Evaluation. Abdom. Radiol..

[290] Wu J., Lv Y., Wang N., Zhao Y., Zhang P., Liu Y., Chen A., Li J., Li X., Guo Y. (2019). The value of single-source dual-energy CT imaging for discriminating microsatellite instability from microsatellite stability human colorectal cancer. Eur. Radiol..

[291] Sun R., Limkin E.J., Vakalopoulou M., Dercle L., Champiat S., Han S.R., Verlingue L., Brandao D., Lancia A., Ammari S. (2018). A radiomics approach to assess tumour-infiltrating CD8 cells and response to anti-PD-1 or anti-PD-L1 immunotherapy: An imaging biomarker, retrospective multicohort study. Lancet Oncol..

[292] Tunali I., Tan Y., Gray J.E., Katsoulakis E., Eschrich S.A., Saller J., Aerts H.J.W.L., Boyle T., Qi J., Guvenis A. (2021). Hypoxia-Related Radiomics and Immunotherapy Response: A Multicohort Study of Non-Small Cell Lung Cancer. JNCI Cancer Spectr..

[293] Zanfardino M., Franzese M., Pane K., Cavaliere C., Monti S., Esposito G., Salvatore M., Aiello M. (2019). Bringing radiomics into a multi-omics framework for a comprehensive genotype-phenotype characterization of oncological diseases. J. Transl Med..

[294] Lafata K.J., Wang Y., Konkel B., Yin F.F., Bashir M.R. (2022). Radiomics: A primer on high-throughput image phenotyping. Abdom. Radiol..

[295] Lenga L., Bernatz S., Martin S.S., Booz C., Solbach C., Mulert-Ernst R., Vogl T.J., Leithner D. (2021). Iodine Map Radiomics in Breast Cancer: Prediction of Metastatic Status. Cancers.

[296] Frix A.N., Cousin F., Refaee T., Bottari F., Vaidyanathan A., Desir C., Vos W., Walsh S., Occhipinti M., Lovinfosse P. (2021). Radiomics in Lung Diseases Imaging: State-of-the-Art for Clinicians. J. Pers Med..

[297] Fusco R., Granata V., Grazzini G., Pradella S., Borgheresi A., Bruno A., Palumbo P., Bruno F., Grassi R., Giovagnoni A. (2022). Radiomics in medical imaging: Pitfalls and challenges in clinical management. Jpn. J. Radiol..

[298] Li Y., Eresen A., Lu Y., Yang J., Shangguan J., Velichko Y., Yaghmai V., Zhang Z. (2019). Radiomics signature for the preoperative assessment of stage in advanced colon cancer. Am. J. Cancer Res..

[299] Gang G.J., Deshpande R., Stayman J.W. (2021). Standardization of histogram- and GLCM-based radiomics in the presence of blur and noise. Phys. Med. Biol..

[300] Muhammad W., Hart G.R., Nartowt B., Farrell J.J., Johung K., Liang Y., Deng J. (2019). Pancreatic Cancer Prediction Through an Artificial Neural Network. Front. Artif. Intell..

[301] Hsieh M.H., Sun L.M., Lin C.L., Hsieh M.J., Hsu C.Y., Kao C.H. (2018). Development of a prediction model for pancreatic cancer in patients with type 2 diabetes using logistic regression and artificial neural network models. Cancer Manag. Res..

[302] Norton I.D., Zheng Y., Wiersema M.S., Greenleaf J., Clain J.E., Dimagno E.P. (2001). Neural network analysis of EUS images to differentiate between pancreatic malignancy and pancreatitis. Gastrointest. Endosc..

[303] Zhu M., Xu C., Yu J., Wu Y., Li C., Zhang M., Jin Z., Li Z. (2013). Differentiation of pancreatic cancer and chronic pancreatitis using computer-aided diagnosis of endoscopic ultrasound (EUS) images: A diagnostic test. PLoS ONE.

[304] Corral J.E., Hussein S., Kandel P., Bolan C.W., Bagci U., Wallace M.B. (2019). Deep learning to classify intraductal papillary mucinous neoplasms using magnetic resonance imaging. Pancreas.

[305] Hussein S., Kandel P., Bolan C.W., Wallace M.B., Bagci U. (2019). Lung and pancreatic tumor characterization in the deep learning era: Novel supervised and unsupervised learning approaches. IEEE Trans Med. Imaging.

[306] Chu L.C., Park S., Kawamoto S., Wang Y., Zhou Y., Shen W., Zhu Z., Xia Y., Xie L., Liu F. (2019). Application of Deep Learning to Pancreatic Cancer Detection: Lessons Learned from Our Initial Experience. J. Am. Coll. Radiol..

[307] Granata V., Fusco R., Belli A., Borzillo V., Palumbo P., Bruno F., Grassi R., Ottaiano A., Nasti G., Pilone V. (2022). Conventional, functional and radiomics assessment for intrahepatic cholangiocarcinoma. Infect. Agent. Cancer.

[308] Xu X., Mao Y., Tang Y., Liu Y., Xue C., Yue Q., Liu Q., Wang J., Yin Y. (2022). Classification of Hepatocellular Carcinoma and Intrahepatic Cholangiocarcinoma Based on Radiomic Analysis. Comput. Math. Methods Med..

[309] Ichikawa S., Isoda H., Shimizu T., Tamada D., Taura K., Togashi K., Onishi H., Motosugi U. (2020). Distinguishing intrahepatic mass-forming biliary carcinomas from hepatocellular carcinoma by computed tomography and magnetic resonance imaging using the Bayesian method: A bi-center study. Eur. Radiol..

[310] Logeswaran R. (2009). Cholangiocarcinoma—An automated preliminary detection system using MLP. J. Med Syst.

[311] Mosconi C., Cucchetti A., Bruno A., Cappelli A., Bargellini I., De Benedittis C., Lorenzoni G., Gramenzi A., Tarantino F.P., Parini L. (2020). Radiomics of cholangiocarcinoma on pretreatment CT can identify patients who would best respond to radioembolisation. Eur. Radiol..

[312] Qin H., Hu X., Zhang J., Dai H., He Y., Zhao Z., Yang J., Xu Z., Hu X., Chen Z. (2021). Machine-learning radiomics to predict early recurrence in perihilar cholangiocarcinoma after curative resection. Liver Int..

[313] Hao X., Liu B., Hu X., Wei J., Han Y., Liu X., Chen Z., Li J., Bai J., Chen Y. (2021). A Radiomics-based approach for predicting early recurrence in intrahepatic cholangiocarcinoma after surgical resection: A multicenter study. Annu. Int. Conf. IEEE Eng. Med. Biol. Soc..

[314] Tang Y., Zhang T., Zhou X., Zhao Y., Xu H., Liu Y., Wang H., Chen Z., Ma X. (2021). The preoperative prognostic value of the radiomics nomogram based on CT combined with machine learning in patients with intrahepatic cholangiocarcinoma. World J. Surg. Oncol..

[315] Li M.D., Lu X.Z., Liu J.F., Chen B., Xu M., Xie X.Y., Lu M.D., Kuang M., Wang W., Shen S.L. (2021). Preoperative survival prediction in intrahepatic cholangiocarcinoma using a ultrasound-based radiographic-radiomics signature. J. Ultrasound. Med..

[316] Park H.J., Park B., Park S.Y., Choi S.H., Rhee H., Park J.H., Cho E.S., Yeom S.K., Park S., Park M.S. (2021). Preoperative prediction of postsurgical outcomes in mass-forming intrahepatic cholangiocarcinoma based on clinical, radiologic, and radiomics features. Eur. Radiol..

